# Sequential PDT and PTT Using Dual‐Modal Single‐Walled Carbon Nanohorns Synergistically Promote Systemic Immune Responses against Tumor Metastasis and Relapse

**DOI:** 10.1002/advs.202001088

**Published:** 2020-07-01

**Authors:** Jingxing Yang, Mengfei Hou, Wenshe Sun, Qinghe Wu, Jia Xu, Liqin Xiong, Yimin Chai, Yuxin Liu, Meihua Yu, Haolu Wang, Zhi Ping Xu, Xiaowen Liang, Chunfu Zhang

**Affiliations:** ^1^ Department of Orthopedics Shanghai Jiao Tong University Affiliated 6th Hospital School of Biomedical Engineering Shanghai Jiao Tong University Shanghai 200030 China; ^2^ Department of Nuclear Medicine Rui Jin Hospital School of Biomedical Engineering Shanghai Jiao Tong University Shanghai 200030 China; ^3^ School of Environment and Biological Engineering Nanjing University of Science and Technology Nanjing Jiangsu 210094 China; ^4^ The University of Queensland Diamantina Institute The University of Queensland Woolloongabba Queensland 4102 Australia; ^5^ Australian Institute for Bioengineering and Nanotechnology The University of Queensland St Lucia Brisbane Queensland 4072 Australia; ^6^ Gallipoli Medical Research Institute Greenslopes Private Hospital Greenslopes Queensland 4120 Australia; ^7^ Department of Biliary‐Pancreatic Surgery Ren Ji Hospital School of Medicine Shanghai Jiao Tong University 800, Dongchuan Road Shanghai 200240 China; ^8^ Department of General Surgery Changzheng Hospital The Second Military Medical University Shanghai 200003 China

**Keywords:** immunotherapy, photodynamic therapy, photoimmunotherapy, photothermal therapy, single‐walled carbon nanohorns, tumor metastases

## Abstract

Immune responses stimulated by photodynamic therapy (PDT) and photothermal therapy (PTT) are a promising strategy for the treatment of advanced cancer. However, the antitumor efficacy by PDT or PTT alone is less potent and unsustainable against cancer metastasis and relapse. In this study, Gd^3+^ and chlorin e6 loaded single‐walled carbon nanohorns (Gd‐Ce6@SWNHs) are developed, and it is demonstrated that they are a strong immune adjuvant, and have high tumor targeting and penetration efficiency. Then, three in vivo mouse cancer models are established, and it is found that sequential PDT and PTT using Gd‐Ce6@SWNHs synergistically promotes systemic antitumor immune responses, where PTT stimulates dendritic cells (DCs) to secrete IL‐6 and TNF‐*α*, while PDT triggers upregulation of IFN‐*γ* and CD80. Moreover, migration of Gd‐Ce6@SWNHs from the targeted tumors to tumor‐draining lymph nodes sustainably activates the DCs to generate a durable immune response, which eventually eliminates the distant metastases without using additional therapeutics. Gd‐Ce6@SWNHs intervened phototherapies also generate durable and long‐term memory immune responses to tolerate and prevent cancer rechallenge. Therefore, this study demonstrates that sequential PDT and PTT using Gd‐Ce6@SWNHs under moderate conditions elicits cooperative and long‐lasting antitumor immune responses, which are promising for the treatment of patients with advanced metastatic cancers.

## Graphical abstract

Gadolinum‐labeled and Ce6‐loaded single wall nanohorns (Gd‐Ce6@SWNHs) are developed for photodynamic and photothermal therapy of breast tumors. Sequential photodynamic and photothermal therapy ablates the primary tumors and triggers synergistic, complementary and long‐lasting host immune response, inhibiting the spontaneous pulmonary metastases and tolerating cancer rechallenge.

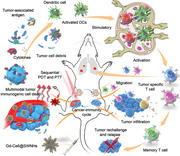

Despite rapid advances in diagnoses and treatments of cancers, tumor metastasis and recurrence are still the main causes of death from malignant tumors globally.^[^[qv: ^1,2^]^]^ Therefore, the ideal tumor therapy not only locally destroys the primary tumors, but also simultaneously triggers the host immune system to recognize and ablate the residual tumor cells at the distant sites. Recently, multifunctional nanoformulations have been intensively explored for tumor‐targeted therapy,^[^[qv: ^3^]^]^ tumor immune microenvironment modulation,^[^[qv: ^4^]^]^ or combination therapies.^[^[qv: ^5,6^]^]^ Among the combination therapies, photoimmunotherapy, including photodynamic therapy (PDT)‐ and photothermal therapy (PTT)‐induced immunotherapy, demonstrates great potentials to treat metastatic tumors.^[^[qv: ^6–10^]^]^ For example, a recent study showed the antitumor efficacy through enhanced immunogenic cell death (ICD)‐associated immunotherapy by PDT and PTT with an oxygen‐delivering hemoglobin, which changed the tumor microenvironment to reverse hypoxia.^[^[qv: ^10^]^]^ However, the immune responses induced by PDT or PTT alone are relatively weak and short‐lived, and they are mostly applied in combination with immune therapeutics such as programmed cell death protein 1/programmed cell death ligand 1 inhibitors and cytotoxic T‐lymphocyte antigen 4 antibody to treat cancer metastases.^[^[qv: ^7,11–15^]^]^ Until now, there is lack of study to directly show the synergistic immunologic responses for effective anticancer treatment especially for cancer metastasis triggered by phototherapy alone.

PDT and PTT have different mechanisms for anticancer treatment. PTT destroys tumor cells by generating heat through light absorption in tumors,^[^[qv: ^16,17^]^]^ while PDT generates reactive oxygen species (ROS) by photosensitizer to kill tumor cells under low‐energy and short‐wavelength (<800 nm) light.^[^[qv: ^18,19^]^]^ Therefore, a combination of PDT and PTT may induce multimodal tumor ICD^[^[qv: ^20^]^]^ and release different types of damage‐associated molecular patterns (DAMPs), tumor‐associated antigens (TAAs), and immunogenicity of cell debris, ^[^[qv: ^17,21,22^]^]^ and enhance the immunogenicity of tumors in situ. On the other hand, combination of PDT and PTT using dual lasers could enhance the therapeutic efficiency of local tumors, increase penetration depth, and enable release and distribution of DAMPs homogeneously and deeply.^[^[qv: ^23,24^]^]^ However, it is still unclear whether the synergistic immunological responses could be achieved by application of PDT and PTT in sequence, and whether the immune responses are strong enough to eliminate tumor metastases and tolerate tumor rechallenge without additional therapeutics.

Carbonaceous materials such as single‐walled carbon nanotubes (SWCNTs)^[^[qv: ^25^]^]^ and graphene oxide (GO)^[^[qv: ^26^]^]^ could induce efficient immune responses for inhibition of cancer metastases. Single‐walled carbon nanohorn (SWNH) may be an alternative material, which is a horn‐shaped carbon nanotube typically with 2–5 nm in width and 40–50 nm in length^[^[qv: ^27^]^]^ with a similar atomic structure. SWNH usually aggregates into flower‐like spherical clusters of 80 to 120 nm in diameter. SWNH has large hydrophobic surface area for drug loading (e.g., chemodrugs, photosensitizers) and broad absorbance in both near‐infrared (NIR) I and II areas, which is optimal for PTT and photoacoustic imaging (PAI) of deeper tissues without the interference of photon scattering.^[^[qv: ^16,23,28^]^]^ Meanwhile, the non‐toxic production process and unique morphology endow SWNHs different nano‐protein and nano‐membrane interplay in pattern compared with SWCNTs and GO,^[^[qv: ^29,30^]^]^ which make SWNHs have the good biocompatibility and biosecurity for in vivo applications. Therefore, SWNHs are an ideal nanovehicle for theranostic agents to enhance phototherapy efficacy in cancers.

Herein, a PDT and PTT combination nanosystem was rationally designed by loading photosensitizer chlorin e6 (Ce6) onto the polymer‐coated SWNHs (**Figure** **1**a). The coordination immune mechanism of PDT/PTT and the role of Gd‐Ce6@SWNHs in the immunity cycle were carefully examined in primary tumors and tumor‐draining lymph nodes (TDLNs) using a bilateral tumor model. Our study indicated that sequential phototherapies of Gd‐Ce6@SWNHs could potently destruct the local primary tumor, release TAAs and DAMPs, and trigger a durable antitumor immune response to effectively eliminate the spontaneous pulmonary metastatic nodules at the early stage. Meanwhile, this therapy strategy elicited a strong and long‐term memory immune response to protect cancer relapse.

**Figure 1:**
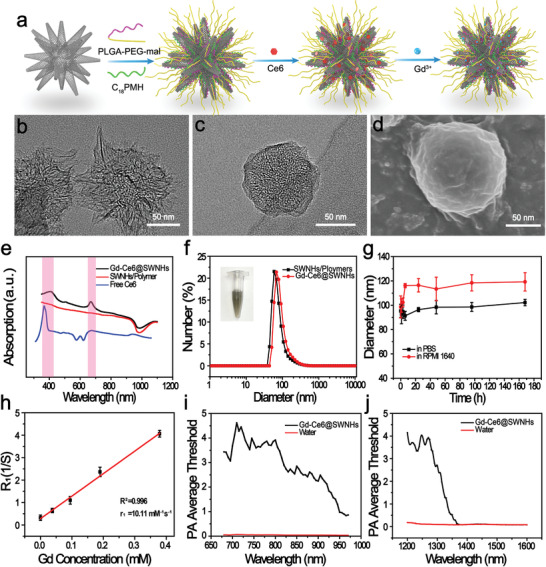
Preparation and characterization of Gd‐Ce6@SWNHs. a) The schematic illustration of Gd‐Ce6@SWNHs preparation. TEM images of b) pristine SWNHs and c) synthesized Gd‐Ce6@SWNHs. d) The scanning electron microscopy (SEM) image of Gd‐Ce6@SWNHs. e) Uv–vis–NIR absorbance spectra of free Ce6, polymers‐coated SWNHs, and Gd‐Ce6@SWNHs, indicating the successful loading of Ce6 onto SWNHs. f,g) Size distribution and stability of Gd‐Ce6@SWNHs in PBS and RPMI‐1640 medium at various incubation times. h) Longitudinal relaxation rate of Gd‐Ce6@SWNHs at different concentrations. Average threshold of photoacoustic signal irradiated by i) NIR‐I lasers (600–1000 nm) and j) NIR‐II lasers (1100–2000 nm).

To improve hydrophilicity of Gd‐Ce6@SWNHs and extend their blood circulation in vivo, we prepared Gd‐Ce6@SWNHs by coating SWNHs with amphiphilic polymers poly (lactide‐*co*‐glycolide)‐*b*‐poly(ethylene glycol)‐maleimide copolymers (PLGA‐PEG‐Mal) and maleic anhydride‐alt‐1‐octadecene (C18PMH) simultaneously through hydrophobic interactions before loading with Gd^3+^ and Ce6.^[^[qv: ^16,31,32^]^]^ Transmission electron microscopy (TEM) images (Figure 1b,c) and Fourier transform infrared spectroscopy spectra (Figure S1a, Supporting Information) of SWNHs and coated SWNHs revealed successful dual amphiphilic polymers surface coating that was dense and smooth (Figure 1d). The size of Cd‐Ce6@SWNH was determined to be 89.5 ± 20.3 nm by measuring the spherical clusters from the TEM imaging (Figure 1c,d). To incorporate photodynamic and magnetic resonance imaging (MRI) properties, Ce6 and Gd^3+^ were loaded onto the polymer‐coated SWNHs sequentially.^[^[qv: ^33^]^]^ The successful loading of Ce6 to SWNHs (Ce6@SWNHs) was unveiled by UV–vis absorbance at 406 and 640 nm (the characteristic peaks for Ce6; Figure 1e). The loading capacity increased with the increase of the Ce6 feeding amount and was saturated at 2 mg mL^−1^. The high Ce6 loading (≈200 wt% SWNHs) may arise from the high hydrophobic surface area of SWNHs (≈281 m^2^ g^−1^; Figure S1c, Supporting Information). After being labeled with Gd^3+^ by chelating Gd ions with porphyrin structures in Ce6 (SWNHs:GdCl_3_ = 1:0.9 wt%), Gd‐Ce6@SWNHs offered strong contrasts in both fluorescence imaging (FI) and MRI. Their hydrodynamic size and zeta potential were 97.3 ± 8.6 nm and −27.8 ± 1.1 mV, respectively (Figure 1f; Figure S1b, Supporting Information). They were stable in PBS and cell culture medium (Figure 1g). The longitudinal relaxivity (*r*_1_) of Gd‐Ce6@SWNHs was determined to be 10.11 mm ^−1^ s^−1^ (Figure 1h), which was 2.9‐fold higher than that of Ce6 (Gd) complex (≈3.51 mm ^−1^ s^−1^).^[^[qv: ^33^]^]^ In addition, Gd‐Ce6@SWNHs is the candidate of multispectral photoacoustic tomography (MSOT) contrast agent since the high thermal performance of SWNHs in NIR‐I region (Figure 1i).^[^[qv: ^16^]^]^ Meanwhile, the strong absorption of SWNHs in NIR‐II region (1200–1300 nm) also rendered the NIR‐II PAI function (Figure 1j). Therefore, this developed Gd‐Ce6@SWNHs could be potentially utilized for multimodal imaging (FI/MRI/PAI/MSOT) guided PTT and PDT dual‐modal cancer therapy.

Next, the photothermal conversion efficiency (*η*) of Gd‐Ce6@SWNHs was determined to be 56.1% with a time constant *τ*_s_ of 132.9 s upon 808 nm laser irradiation (Figure S2a,b, Supporting Information), which was superior to their counterparts (SWCNT, ≈30%^[^[qv: ^32,34^]^]^ and GO, ≈25%)^[^[qv: ^35,36^]^]^ reported previously. Consistent with other PTT agents,^[^[qv: ^33,36,37^]^]^ the photothermal performance of Gd‐Ce6@SWNHs also showed concentration‐, laser power density‐ and irradiation time‐dependent manners (Figures S2c,d and S3a, Supporting Information). Moreover, Gd‐Ce6@SWNHs were photothermally stable where the temperature of Gd‐Ce6@SWNHs suspension could still recover to its initial maximum level (Δ*T* ≈ 37 °C) in each cycle and no aggregations were observed after irradiation for five cycles (Figures S2c and S3b, Supporting Information). Impressively, unnoticeable Ce6 was released from Gd‐Ce6@SWNHs as detected by UV–vis absorbance spectra after hyperpyrexia treatments.

To further assess the photothermal effects of Gd‐Ce6@SWNHs on cells, breast cancer cells (4T1) were co‐incubated with Gd‐Ce6@SWNHs (10 µg mL^−1^) for 12 h, followed by irradiation with an 808 nm laser (1.5 W cm^−2^) for different time periods. Calcein‐AM and propidium iodide (PI) were used to stain the cells to indicate the live and dead cells (**Figure** **2**a). Cell death appeared after irradiation for 3 min, and was apparent after irradiation for 5 min, and no living cells were detected after irradiation for 7 min. In contrast, no cytotoxicity of Gd‐Ce6@SWNHs was observed for cells treated at a high concentration (100 µg mL^−1^ in SWNHs) for 72 h (Figure S4, Supporting Information). Consistent with previous reports where thermal treatment‐induced cell death by Gd‐Ce6@SWNHs was concentration‐, laser power density‐ and irradiation time‐dependent (Figure 2b,c),^[^[qv: ^8,36,38^]^]^ Gd‐Ce6@SWNHs showed a high thermal therapeutic effect on cancer cells.

**Figure 2:**
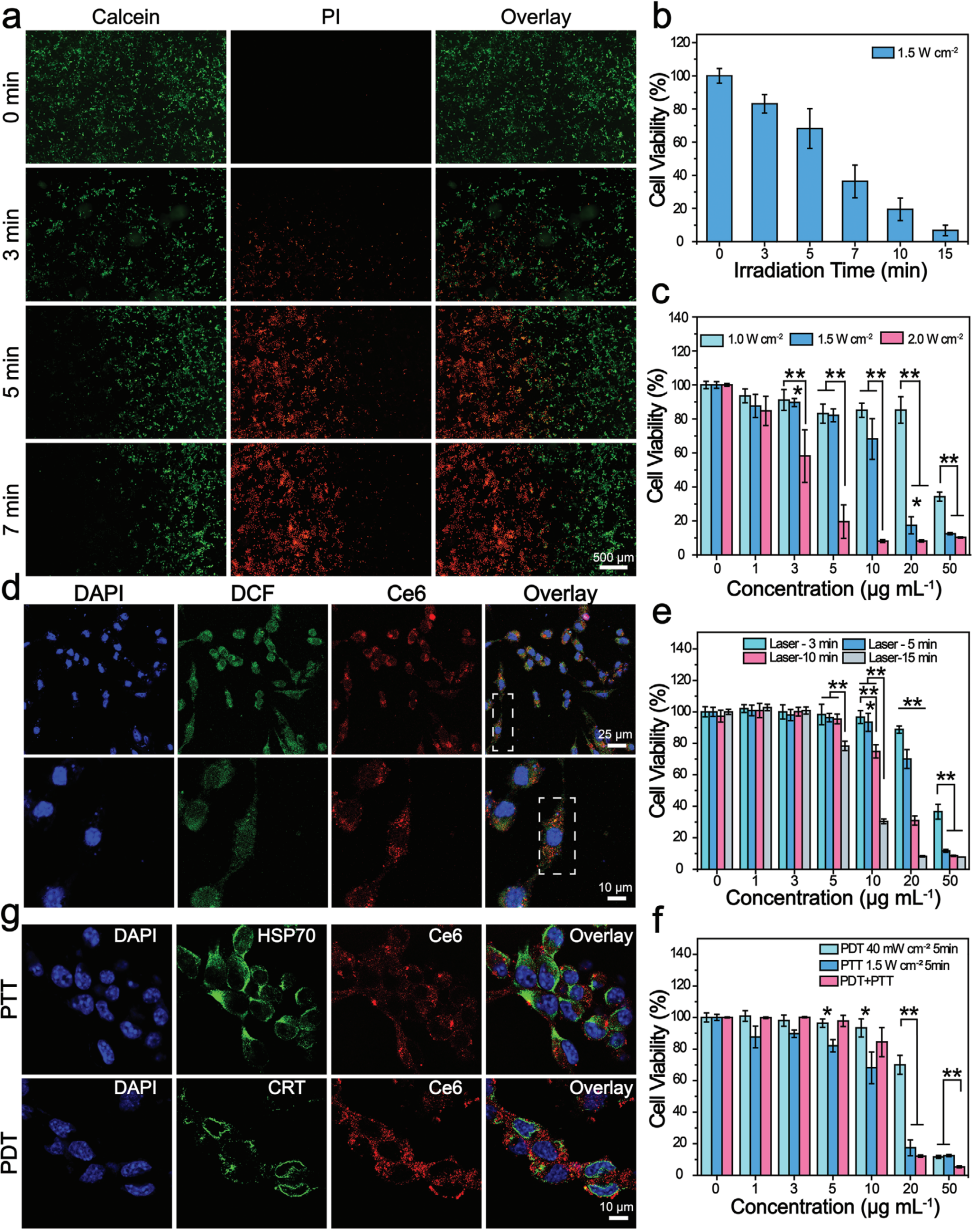
Phototherapeutic effects and immunological responses induced by phototherapy in vitro. a,b) Cell viability of 4T1 cells incubated with Gd‐Ce6@SWNHs (10 µg mL^−1^) after PTT with an 808 nm laser (1.5 W cm^−2^) for 3, 5, or 7 min. Cell viability imaging by fluorescence microscopy after staining with the live‐dead cell staining kit (Calcein‐AM/PI) (a) and quantified by CCK‐8 assay (b). c) Cell viability of 4T1 cells incubated with Gd‐Ce6@SWNHs at different concentrations and illuminated under an 808 nm laser for 5 min at different density (1.0, 1.5, and 2 W cm^−2^). d,e) PDT applied to Gd‐Ce6@SWNHs treated 4T1 cells. d) Detection of intracellular singlet oxygen of 4T1 cells treated with Gd‐Ce6@SWNHs (10 µg mL^−1^) for 12 h and illuminated with a 650 nm laser for 5 min at the density of 40 mW cm^−2^ (upper row). Magnified images of selected regions from (d) to perceptibly locate the nanovehicles and intracellular reactive oxygen (lower row). e) Cell viability of 4T1 cells incubated with Gd‐Ce6@SWNHs at different concentrations (1, 3, 5, 10, 20, and 50 µg mL^−1^) and illuminated with a 650 nm laser for various time intervals (3, 5, 10, and 15 min) at the density of 40 mW cm^−2^. f) PDT and PTT sequential therapy of 4T1 cells treated with Gd‐Ce6@SWNHs and illuminated with a 650 nm at the density of 40 mW cm^−2^ and subsequently under an 808 nm at the density of 1.5 W cm^−2^ for 5 min. g) Immunofluorescence staining of HSP 70 and CRT as a consequence of immunogenic cell death (ICD) mediated by PTT or PDT. All values are expressed as mean ± SD. Statistical significance (*p*) was calculated by one‐way analysis of variance (ANOVA) using the Tukey′s post‐test; *p*‐value: *p < 0.05; **p < 0.01.

PDT effect of Gd‐Ce6@SWNHs on 4T1 cells was also examined by detecting ROS production using 2,7‐dichlorodi‐hydrofluorescein diacetate based assay.^[^[qv: ^39,40^]^]^ After cells were treated with Gd‐Ce6@SWNHs and illuminated by a 650 nm laser (40 mW cm^−2^), fluorescence signals of Ce6 in cell cytoplasm and ROS generation were observed (Figure 2d,e), and the latter was increased with the prolongation of irradiation time (Figure S5, Supporting Information). As a result, the cell viability decreased from 96.6 ± 4.1% to 30.4 ± 1.4% after irradiation for 3 and 15 min, respectively (Figure 2e). Moreover, cell death was Gd‐Ce6@SWNHs concentration‐dependent where cell viability dramatically dropped to 7.7 ± 0.8% after incubation with 50 µg mL^−1^ Gd‐Ce6@SWNHs. These observations indicated that Gd‐Ce6@SWNHs also had a good photodynamic therapeutic effect in cancer cells.

Motivated by the effective PTT or PDT of Gd‐Ce6@SWNHs for cancer cells, we subsequently examined the synergistic therapeutic effect of PDT and PTT in vitro. 4T1 cells were co‐incubated with various concentrations of Gd‐Ce6@SWNHs for 12 h, irradiated with 650 nm laser (40 mW cm^−2^) and 808 nm laser (1.5 W cm^−2^) for 5 min in sequence. Similar to PTT or PDT alone, combination therapy was Gd‐Ce6@SWNHs concentration‐dependent (Figure 2f). Cell viability decreased from 99.8 ± 0.5% to 5.3 ± 0.6% when Gd‐Ce6@SWNH concentrations increased from 1 to 50 µg mL^−1^ by PDT and PTT in sequence. However, compared to single PDT or PTT, the combination therapy did not show higher potency against cancer cells at lower concentrations (below 10 µg mL^−1^). At higher concentrations of Gd‐Ce6@SWNHs, both the single therapy and combination therapy induced cell death markedly, but PTT dominated combination therapy. To explore the underlying mechanism, we evaluated the half‐maximal inhibitory concentrations (IC_50_) of PDT, PTT, and PDT + PTT combination treatment, which were 43.47, 14.11, and 13.75 µg mL^−1^, respectively (Figure S6, Supporting Information). The combination index (CI) of PDT + PTT was then calculated to be 1.29 (CI > 1), indicating the antagonistic therapeutic effect of PDT and PTT on tumor cells,^[^[qv: ^16,41^]^]^ which was different from highly synergistic therapeutic effect of the combined phototherapies, as reported previously.^[^[qv: ^19,42^]^]^ This discrepancy may arise from different nanoformulations, treatment conditions, and the sequence of PDT and PTT applied for the therapy. Moreover, the thresholds of cell death by PDT and PTT treatment may also be different, and a single treatment modal may dominate the combination therapy under a particular treatment condition.

As mild phototherapy may damage tumor cells through the generation of stress responses to change the extracellular microenvironment,^[^[qv: ^20,22^]^]^ damaged tumor cells could secrete DAMPs to protect themselves from the therapy. Furthermore, a fever‐ranged temperature of 39–41 °C could activate potential antitumor immune responses by altering membrane characteristics and denaturation of proteins and biomolecules.^[^[qv: ^17,21^]^]^ Therefore, these tumor‐derived DAMPs induced by PDT + PTT combination treatment may act as signals to protect tumor cells from therapies,^[^[qv: ^9,21,43^]^]^ and are also regarded as immune stimulators to enhance antigen uptake by the antigen‐presenting cells (APCs) and activate adaptive immunity to secrete immune cytokines.^[^[qv: ^13,20,21,43^]^]^ To confirm our speculation, immunostimulatory molecules were examined after the combination therapy, including endoplasmic reticulum chaperone calreticulin (CRT) and heat shock protein (HSP) 70. As shown in immunofluorescence images (Figure 2g), both phototherapies generated or activated specific stress response proteins. Moreover, HSP 70 was synthesized in the nucleus and continuously migrated to the cell membrane after the treatment as detected by fluorescence imaging (Figure S7, Supporting Information). Exposure of these immune proteins was favorable for immune response triggered by phototherapies in vivo.

We further found PDT + PTT using Gd‐Ce6@SWNHs stimulated dendritic cells (DCs) maturation in vitro (Figure S8, Supporting Information). To understand the immunogenicity of nanovehicle, we first studied the effects of Gd‐Ce6@SWNHs on DCs maturation. Gd‐Ce6@SWNHs provoked the upregulation of co‐stimulatory molecules CD80/CD86 in DCs and the population of mature DCs (mDCs) increased from 19.9% to 58.6% when the dose increased from 5 to 20 µg mL^−1^ (Figure S8a, Supporting Information). The immunogenic effects of Gd‐Ce6@SWNHs on DCs were analyzed by different gene expression of immune‐related proteins, where the obvious upregulation of TNF‐*α*, IL‐6, CD80, and IL‐1*β* were observed in Gd‐Ce6@SWNHs‐treated DCs without phototherapy (Table S1, Supporting Information). In contrast, there was no obvious immunogenicity of PEG, Ce6, or GdCl_3_ on DCs and mDCs were almost undetectable. These results indicate that SWNHs acted as an immune adjuvant.

PTT and PDT induced upregulation of HSP 70 and CRT in tumor cells, and these DAMPs could activate DCs to present the TAAs to naive T cells.^[^[qv: ^20,21^]^]^ The combination of PDT and PTT induced higher population of mDCs (79.4%) than single PDT (45.9%) or PTT (48.5%) using a transwell system (Figures S8b and S9, Supporting Information). This augment may arise from the fact that PDT and PTT induced ICD of tumor cells in different patterns, generating distinct stimulators to synergistically activate more DCs. Most immune‐related genes were significantly upregulated by combination therapy of PTT and PDT compared to a single therapy, especially for IL‐6, IFN‐*γ*, and CD80 (Table S1, Supporting Information). These cytokines and molecules are typical markers of adaptive immunity for positively mediating the immune cells.^[^[qv: ^12,44,45^]^]^ In contrast, PTT mainly stimulated DCs to secrete IL‐6 and TNF‐*α*, while PDT mainly upregulated IFN‐*γ* and CD80. Therefore, PDT and PTT tandem treatments on tumor cells induced diverse stimulations on DCs, leading to more mature DCs. These results suggest that the combination of PDT + PTT displayed a synergistic and complementary effect on activating immature DCs (iDCs) by Gd‐Ce6@SWNHs‐based phototherapies.

The biodistribution and tumor‐targeting efficiency of Gd‐Ce6@SWNHs were evaluated by multimodal imaging. Due to the prominent photothermal performance of SWNHs in the NIR‐I region, MSOT was first conducted in mice of orthotopic breast cancers which were intravenously injected with Gd‐Ce6@SWNHs at a dosage of 10 mg kg^−1^ (**Figure** **3**a). Tumor and its adjacent tissues were reconstructed by a 3D model in real‐time images (Figure S10a and Videos S1–S8, Supporting Information). As shown in MSOT, Gd‐Ce6@SWNHs were mainly distributed in tumor blood vessels at 1 h post‐injection, and gradually extravasated and penetrated into tumor parenchyma. Consistent with MSOT imaging, the signal intensity of SWNHs in the tumor area gradually increased over time and reached the maximum signal of 375 times at 6 h post‐injection (Figure S11a, Supporting Information). The high tumor‐targeting efficiency of Gd‐Ce6@SWNHs was also verified by MRI (Figure S10b, Supporting Information). In addition to efficient absorbance at NIR‐I region, SWNHs also have a strong absorbance at the NIR‐II window (1200–1300 nm), in which the biological tissues are transparent.^[^[qv: ^31^]^]^ We performed PAI at 1100–1900 nm wavelength to study tumor penetration and intratumor distribution of Gd‐Ce6@SWNHs without the tissue background (Figure 3b). Consistent with MSOT observations, Gd‐Ce6@SWNHs were mainly distributed in the peripheral blood vessels of the tumor after 1 h injection, gradually permeated into deep tissues and unevenly distributed into tumor parenchyma rather than peripheral blood vessels. The high tumor targeting of Gd‐Ce6@SWNHs is mainly through the enhanced permeability and retention effect as the vascular system in tumor tissues is completely different from blood vessels in normal tissues.^[^[qv: ^46^]^]^ Gd‐Ce6@SWNHs have unique morphology (dahlia‐like aggregation), appropriate size of 80–120 nm, multi‐branched structures, and long blood half‐life, which contribute to the high tumor targeting efficiency of Gd‐Ce6@SWNHs. The uneven distribution may arise from tumor heterogeneity or necrosis.^[^[qv: ^47^]^]^ Therefore, Gd‐Ce6@SWNHs were found to efficiently target and permeate deeply into tumors, which is different from other sp^2^‐bonded carbon materials such as SWCNTs^[^[qv: ^48^]^]^ and GO.^[^[qv: ^38^]^]^ The in vivo biodistribution of Gd‐Ce6@SWNHs further confirmed efficient tumor accumulation by 3D MSOT (Figure 3c; Figure S12, Supporting Information). The fluorescence imaging of ex vivo excised organs and tumors showed a higher concentration of Ce6@SWNHs in the tumor as well as in reticuloendothelial system (Figure 3e; Figure S13, Supporting Information), indicating they were mainly sequestered by the reticuloendothelial system and metabolized through the hepatobiliary routes for the most nanoformulations.^[^[qv: ^37,48–50^]^]^ As a consequence, high concentrations of Gd‐Ce6@SWNHs in the liver and spleen led to Ce6 fluorescence quenching, being invisible in ex vivo fluorescence imaging (Figure 3e; Figure S13d, Supporting Information). The blood half‐life of Gd‐Ce6@SWNHs was 18.9 h determined by PA imaging for 48 h and fitted by a two‐compartment model (Figure 3d).^[^[qv: ^51^]^]^ Furthermore, after injection for 7 days, SWNH nanosystem was still visualized in TDLNs (Figure 4i), indicating that Gd‐Ce6@SWNHs was able to migrate from the tumor to the lymph nodes and retain there for a long time. This particle retention is beneficial to mature APC and activate cytotoxic T lymphocytes (CTLs).

**Figure 3:**
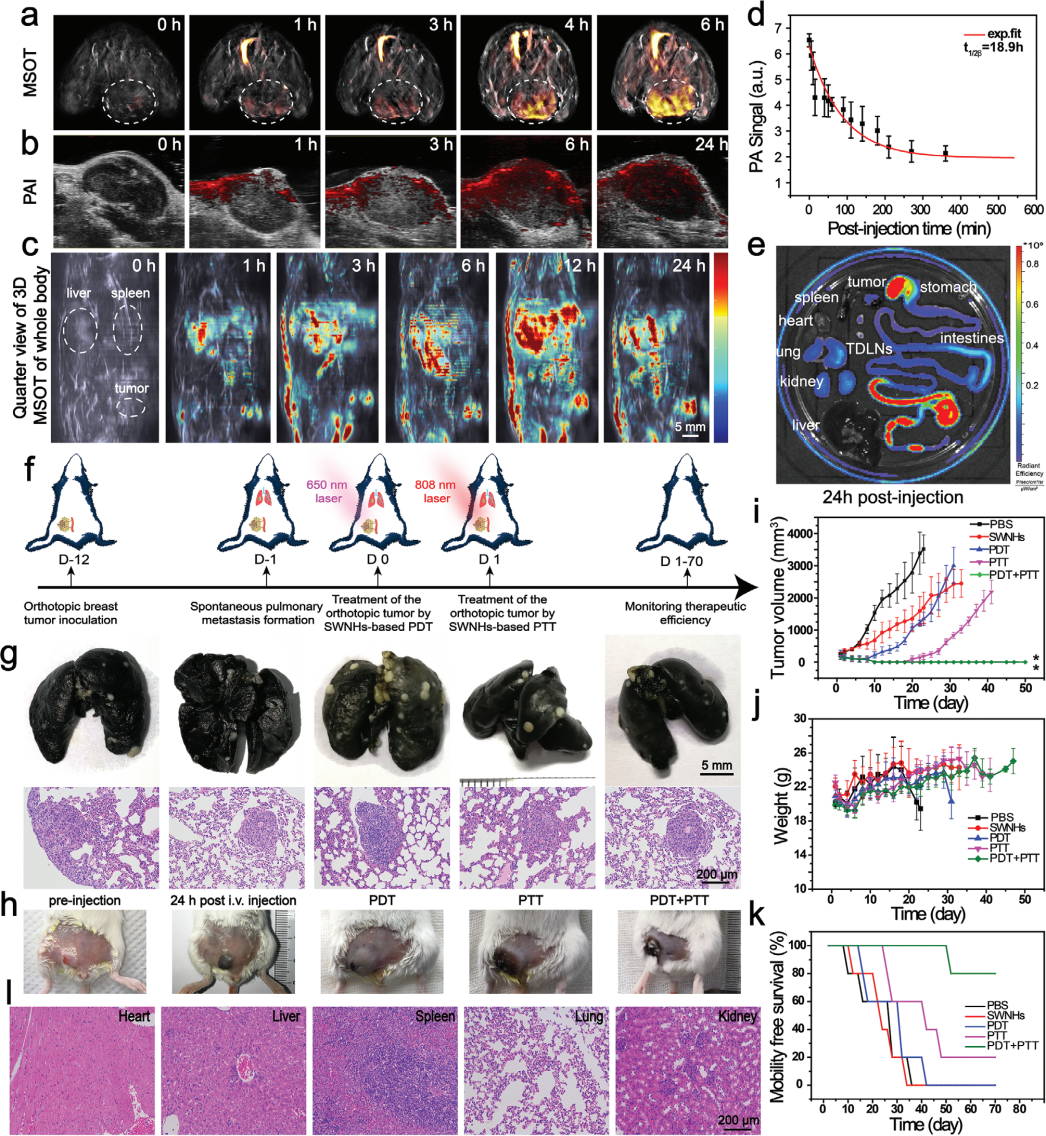
In vivo therapeutic efficiency of Gd‐Ce6@SWNHs on orthotopic breast cancer tumors. a,b) PAI of orthotopic breast cancer models after intravenously administered with Gd‐Ce6@SWNHs at the dosage of 10 mg SWNHs per kg b. w. MSOT was performed under the NIR‐I region (680–980 nm) (a) and PAI at the NIR‐II window (1100–1900 nm) (b). c) In vivo real‐time visualization of Gd‐Ce6@SWNHs in tumor‐bearing mice by 3D MSOT. Key organs (e.g., liver, spleen, and tumor) were indicated by the dotted circles. d) Half‐life of Gd‐Ce6@SWNHs was determined by fitting PA signals in the internal jugular vein based on a two‐compartment model. e) Biodistribution of Gd‐Ce6@SWNHs after intravenous injection in tumor‐bearing mice for 24 h. Fluorescence imaging of ex vivo major organs and tumors at the emission of Ce6. f) Schematic illustration of establishment of orthotopic breast cancer model and spontaneous pulmonary metastasis and strategy of sequential PDT + PTT. g) Identification of spontaneous pulmonary metastases by India ink perfusion (upper row) and H&E staining of lung slice (lower row) before treatments. h) Photographs of tumors before and after Gd‐Ce6@SWNHs injection without phototherapy as well as tumors after Gd‐Ce6@SWNH injection and subsequently treated by PDT, PTT, or PDT + PTT, respectively. i–k) Evaluation of therapeutic efficacy of phototherapy in tumor‐bearing mice treated with Gd‐Ce6@SWNH. Tumor growth curves (i), weight changes (j), and mobility free survival rate (k) of 4T1 tumor‐bearing mice were recorded for 70 days post‐treatment. l) H&E staining of the major organs include heart, liver, spleen, lung, and kidney of the PDT + PTT group at 70 days post‐treatment; *p < 0.05; **p < 0.01.

**Figure 4:**
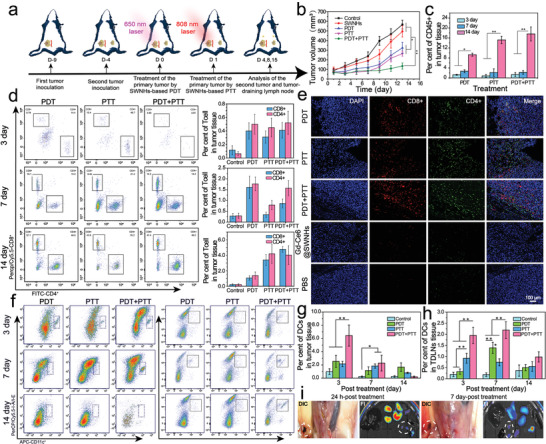
Evaluation of the immune abscopal effect for inhibiting the growth of distant tumors. a) A schematic illustration of the development of a bilateral 4T1 tumor model and strategies for phototherapy and immune analysis. b) Growth curves of distant tumors after the treatment for the primary tumor. c) Proportions of tumor‐infiltrating of CD45+ cells in the distant tumors after various therapies for the primary tumors. d) The representative flow cytometric analysis of tumor‐infiltrating T cells (CD4+ and CD8+) in distant tumors and their relative proportions in tumor tissues at 3, 7, and 14 days from different treatment groups. e) Representative immunofluorescence images of infiltrating T lymphocytes in the distant tumor at 14 days post‐treatment in different treatment groups. f) Recruitment of DCs into the distant tumor (left columns) and tumor‐draining lymph nodes (right columns) in different treatment groups at various time points after treatments. g,h) Proportions of DCs (Live CD45+CD11c+MCH II+CD80+CD86+) in the distant tumor (g) and tumor‐draining lymph nodes (h) at day 3, 7, and 14 after different phototherapy. i) Visualization and fluorescence images of long‐term migration of Gd‐Ce6@SWNHs to tumor‐draining lymph nodes at 24 h and 7‐day post PDT + PTT combination treatment. The lymph nodes were indicated by the circles; *p < 0.05; **p < 0.01.

We then explored the combination therapy for simultaneously inhibiting the primary tumors and their pulmonary metastases. A spontaneous lung metastasis model from orthotopic breast cancer (4T1) was established (Figure 3f,g). The optimal irradiation conditions were determined by real‐time monitoring the temperature of the tumor site post intravenous injection of Gd‐Ce6@SWNHs (10 mg kg^−1^ b.w) for 24 h (Figure S14, Supporting Information). Consistent with previous reports, the tumor temperature elevation was laser power intensity‐ and irradiation time‐dependent.^[^[qv: ^8,16^]^]^ Since PDT and PTT have different mechanisms to induce cell death and trigger immune cell death in different patterns, we performed PDT then followed by PTT in order to trigger more diverse immune cell death (Figure 3f). We exposed tumors to the wavelength of 650 nm for 10 min with a low laser power (40 mW cm^−2^) for PDT. During PDT performance, there was unnoticeable temperature change, therefore, tumors were not overheated and killed by hyperthermia. After PDT, the tumors were irradiated with an 808 nm laser at the power intensity of 0.5 W cm^−2^ for 10 min for PTT and the temperature was controlled around 53 °C, according to the previous reports.^[^[qv: ^8,52^]^]^

Tumor accumulation of Gd‐Ce6@SWNHs was obvious by visual inspection over 3 h post‐injection (Figure S15, Supporting Information). Tumors became puffy after PDT and further shrunk after PTT (Figure 3h), suggesting the different ICD patterns induced by PDT and PTT. Following the treatment, the tumor size and survival of the mice were monitored (Figure 3i–k). Interestingly, compared to the PBS group, tumor growth was markedly suppressed in Gd‐Ce6@SWNHs treatment (Figure 3i). This may arise from the fact that Gd‐Ce6@SWNHs are an immunologic adjuvant and stimulate the systemic immune response against tumors, as mentioned above (Figure 2g; Figure S8, Supporting Information). For either single phototherapy (PDT or PTT) or combination therapy, the growth of the treated tumors was immediately detained after the laser irradiation and completely ablated at 5 days post‐treatment. Unfortunately, tumor relapse was observed at 7th day and 20th day for PDT or PTT group of mice, respectively, and consequently, these mice had shorter life spans (about 16–50 days). In sharp contrast, no recurrence was observed in the PDT + PTT combination group and 80% of treated mice survived for 70 days.

To examine the possible inhibition of lung metastases by Gd‐Ce6@SWNHs mediated PDT + PTT in vivo, three mice in combination therapy group were euthanized at 70 days post‐treatment, and major organs (i.e., heart, liver, spleen, lung, and kidney) were harvested and examined histologically (Figure 3l). As shown in H&E staining, lung metastases vanished and no damage was found in normal organs. In our study, in addition to the primary tumors, spontaneous pulmonary metastases were also eliminated after combination therapy (80% of mice), and no recurrence occurred even during the next 18 months post‐treatment. Typically, locally applied NIR phototherapy is only able to inhibit the primary tumors that directly illuminated, but cannot treat their metastases at distant sites. As recent studies revealed that both PDT and PTT could evoke host immune responses, but the responses were weak and unendurable.^[^[qv: ^7,9,11,14,43^]^]^ This potent therapeutic performance may be ascribed to the SWNHs‐mediated high efficient phototherapies and synergistic immune responses. With further assistance of SWNHs as an immune adjuvant, the pulmonary metastasis was successfully suppressed by the strong immune abscopal effect. Moreover, blood routine examinations at 6, 9, and 18 months after the treatment (Figure S16, Supporting Information) showed all the blood routine indexes were normal, suggesting that Gd‐Ce6@SWNHs were no appreciable systemic toxicity in vivo.

PDT + PTT treatment on pulmonary metastases was further assessed if immune abscopal effect induced by combination phototherapy using a bilateral tumor model (**Figure** **4**a). Compared to the PBS group, the growth of the second tumors was significantly suppressed after the primary tumors were treated by PDT, PTT, or PDT + PTT, where the combination therapy was the most potent (Figure 4b) and tumor growth was delayed by 1.75‐, 2.14‐, and 4.20‐fold in terms of the volume (*p* < 0.01) at 14 days post‐treatment, respectively. To investigate the mechanism of inhibiting metastasis, we analyzed the major immune cells in the second tumors. First, the population of leukocytes (CD45+) continuously increased, indicating the improvement of tumor inflammation after the treatment (Figure 4c).^[^[qv: ^53^]^]^ Tumor infiltrated CD8+ T cells (gating on Live CD45+CD3+CD4‐CD8+; Figure S17, Supporting Information), the direct evidence of systemic immunity,^[^[qv: ^22^]^]^ were examined at 3rd, 7th, and 14th day after the primary tumors were subjected to phototherapies (Figure 4d). Flow cytometry analysis revealed that PDT increased CD8+ T cells from 0.4% at 3rd day to 1.61% at 7th day post‐treatment, and then declined to 1.07% at 14th day. Whereas, the immune response triggered by PTT was rather delayed. An obvious increase in CD8+ was observed at 14 days post‐treatment (3.39%). However, unlike that by PTT or PDT alone, CD8+ T cells in the combination treatment group increased steadily from 0.41% at 3 days to 4.77% at 14 days post‐treatment, indicating the induction of a rapid and long‐lasting immunity. In addition to CD8+ T cells, CD4+ T cells (Live CD45+CD3+CD4+CD8‐), a key immune T helper cell, showed similar infiltration trend to that of CD8+ T cells in the respective treatment groups. The infiltration of CD8+ and CD4+ T cells in the distant tumors were further confirmed by immunohistological staining. As shown in Figure 4e, massive infiltration of CD8+ and CD4+ T cells were observed in the second tumors after the primary tumors were treated with phototherapies. There were only marginal CD8+/CD4+ T cells detected in Ce6‐Gd@SWNHs and PBS groups. These observations demonstrate that both single PDT and PTT could trigger host immune responses, but the induction was at a different pace and not strong enough to suppress the metastatic tumors. Sequential applications of PDT and PTT after i.v. administration of Gd‐Ce6@SWNHs evoked durable immune effects, inhibiting sufficiently the growth of the remote tumors at the early stage.

To give further insight into the activation process of immune response triggered by phototherapies in vivo, we then assessed the population and status of DCs in the second tumor and TDLNs after the treatments (Live CD45+CD11c+MCH II+CD80+CD86+; Figure S17, Supporting Information), which were prerequisites for induction of adaptive immunity.^[^[qv: ^54^]^]^ Ascribed to quick responses to phototherapy, the infiltration of DCs in the distant tumors reached 2.54% (PDT), 2.15% (PTT), and 6.44% (PDT + PTT) in a short term (3 days post‐treatment), respectively, and then gradually declined within 14 days post‐treatment (Figure 4f,g). Correspondingly, the activated DCs were increased to 80% (PDT), 81.5% (PTT), and 57.6% (PDT + PTT), and then fell to 11.9%, 51.2%, 53.0%, respectively (Figure S18, Supporting Information). Interestingly, the recruitment of DCs to TDLNs showed distinct trends in different treatment groups. The PDT + PTT recruited more DCs than PDT or PTT alone at each time point (Figure 4h) and they were maintained at a high level in TDLNs within 7 days post‐treatment (Figure 4i). More DCs drained to lymph nodes meant that more tumor‐specific T cells could be activated.^[^[qv: ^12,54^]^]^

Activation of the host immune system triggered by phototherapies includes the following steps. Abundant iDCs are first rapidly recruited into tumors via immune factors secreted by the stress cells in the primary tumor after light irradiation.^[^[qv: ^7,12,55^]^]^ Subsequently, tumor‐infiltrating DCs are stimulated to mDCs by a variety of immunogenic substances, such as DAMPs, TAAs, and cell debris. They then migrate to the lymph nodes, in which the naive T cells are activated to effector T cells (CD8+ and CD4+), and return to the primary tumor and distant metastases owing to the tumor‐homing effect induced by tumor‐specific antigens. However, to achieve the long‐lasting immune response triggered by phototherapy, it is necessary to maintain the immune cycle with multi‐rounds or sustainable stimulation. As demonstrated in this study, sequential PDT + PTT combination therapy on Gd‐Ce6@SWNHs‐treated tumor cells generated a more diverse ICD than single PDT or PTT, which stimulated more DCs to mature, as revealed by flow cytometry data. As shown in Figure 4i, Ce6‐Gd@SWNHs absorbing immunogenic substances released from the dying tumor cells could be continuously drained to TDLNs after the treatments and stay there for a long time, further boosting the host immune response and inducing durable stimulation of DCs via the immune cycle.^[^[qv: ^55^]^]^ As a result, multiple immune‐related factors (TNF‐*α*, IFN‐*γ*, IL‐*α*, IL‐1*β*, IL‐2, IL‐6, and IL‐12) elevated and maintained in peripheral blood at a high level after the combination therapy (Figure S19, Supporting Information).

As immunological memory is an important factor in adaptive immune response system to battle diseases when the same subtype of pathogens invade again,^[^[qv: ^12^]^]^ we examined the effects of combination of PDT and PTT on long‐term immunological memory. Firefly luciferase stably expressed 4T1 (fLuc‐4T1) cells were i.v. injected into three cured mice at 50‐day post‐treatment and monitored using the bio‐fluorescence imaging system (**Figure** **5**a). The rechallenged fLuc‐4T1 tumor cells were completely disappeared in the mice with orthotopic tumors ablated by the combined phototherapy at 2 weeks post‐injection. In sharp contrast, the mice that experienced surgery or just PBS injection had no appreciable inhibitory effect on the rechallenged cells, and eventually, no mice survived for the next 6 weeks. Consistent with FI observations in vivo, massive metastatic nodules were distinguished in the lungs from PBS or surgery group by visual inspection and H&E staining, while no nodules were found in the combination therapy group (Figure 5b). In addition, no obvious histopathological abnormality or lesions were found in these organs, especially for no metastatic nodule of lungs and TDLNs even at 18 months post‐treatment (Figure 5c). Furthermore, all of the measured blood biochemistry parameters and complete blood panel data of PDT + PTT treated group appeared to be normal at different time points (Figure S16, Supporting Information). In conclusion, no appreciable systemic toxicity was observed in vivo after treatment by Gd‐Ce6@SWNHs and combination of PDT + PTT at our experimental dosage as long as 18 months post‐treatment.

**Figure 5:**
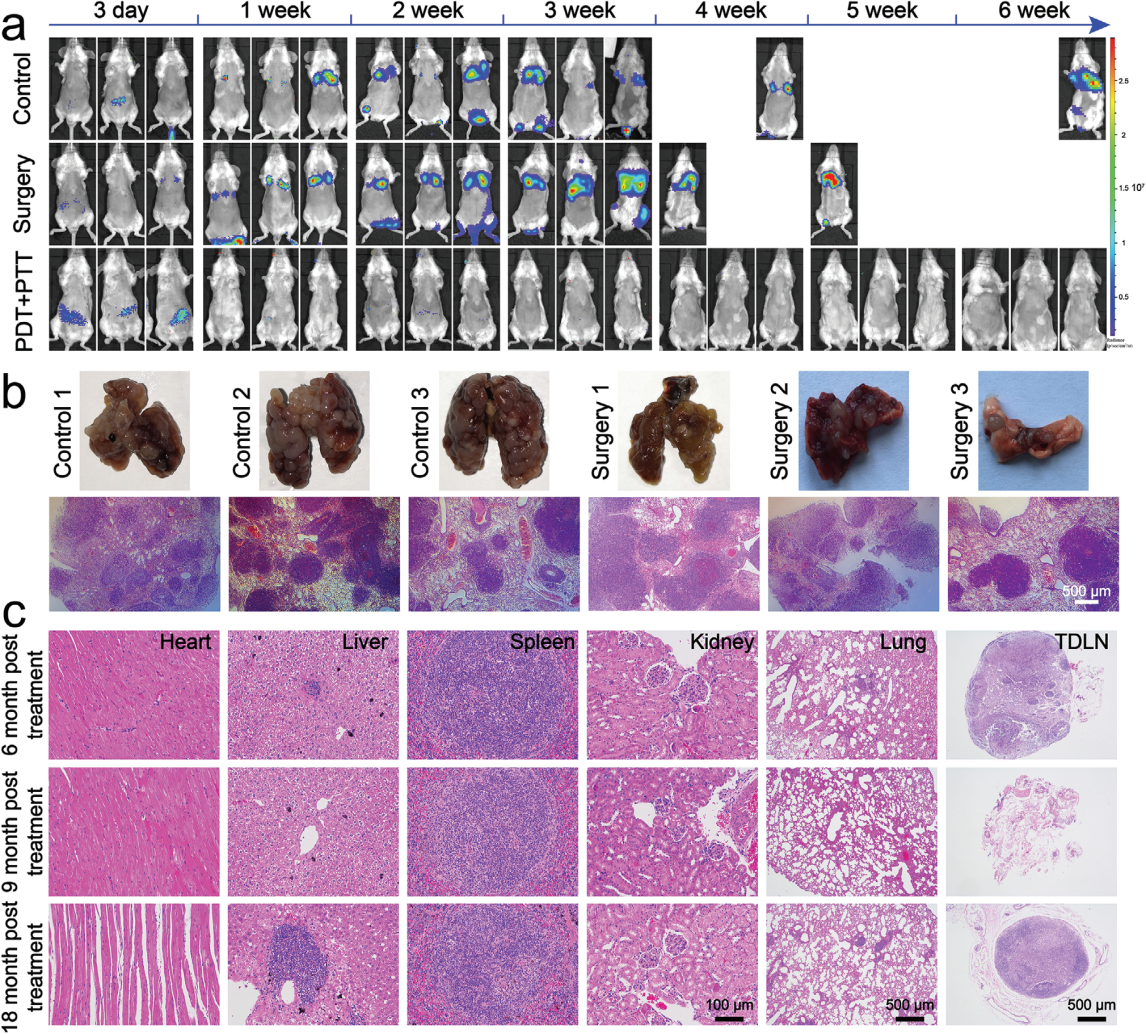
Long‐term immune‐memory effects of Gd‐Ce6@SWNHs after phototherapy in tumor‐bearing mice. a) Biofluorescence imaging of the cured mice rechallenged by fluorescein‐transfected 4T1 cells (2 × 10^5^). b) Examination of pulmonary metastases in all groups. c) Long‐term systematic toxicity of Gd‐Ce6@SWNHs in vivo evaluated by H&E staining of major organs and lymph nodes after 6, 9, and 18 months of fLuc‐4T1 rechallenge.

PDT and PTT using various nanoformulations have been extensively explored in the preclinical studies as the novel and promising treatment strategies.^[^[qv: ^9,21,22^]^]^ Both PDT and PTT not only destroy tumor cells through the different mechanisms but also induce immunogenic tumor cell death to initiate a systemic antitumor immune response. However, due to the limited penetration of light, only partial tumors could respond to the stimulation, resulting in inadequate therapeutic efficiency of the primary tumor and insufficient immune responses for tumor metastases.^[^[qv: ^11,14,25,43^]^]^ One possible reason was that single phototherapy was unable to elicit abundant ICDs from tumor cells. On the other hand, the lack of long‐term stimulation of DCs may also limit the efficiency of autoimmunity. In the current study, an SWNH based theranostic nanoplatform was developed by combining drug carriers and immune adjuvant for PDT and PTT, eliminating the primary tumors by tandem and mild phototherapies and simultaneously evoking durable host immunity to inhibit their metastases in deep tissues. The excellent therapeutic performance is achieved by Gd‐Ce6@SWNHs‐mediated phototherapy and enhanced host immune responses attributed to the following mechanisms (**Figure** **6**). First, Gd‐Ce6@SWNHs could efficiently accumulate in the tumors. Subsequently, sequential PDT and PTT eliminate primary tumor through different mechanisms to induce tumor cell apoptosis (ROS induced by PDT) or necrosis (heat generation by PTT). Second, the combination of PDT with PTT induced more diverse immune cell death and TAAs compared to single PDT or PTT due to different penetration depth and treatment mechanisms of PDT and PTT. The combination of PDT and PTT using dual lasers could increase penetration depth in tissues, and enable release and distribution of DAMPs homogeneously and deeply. The diverse antigens released from the dying tumor cells after PDT and PTT could be absorbed on SWNHs in situ and was prone to be recognized and taken up by iDCs, leading to efficient maturation of DCs. Third, we performed PTT one day after PDT to ensure the sufficient release of DAMPs and TAAs in deep tissues from the injured cells after PDT. Fourth, SWNHs‐carrying tumor‐derived antigens could continuously migrate from tumor sites to lymph nodes and stay for a long time. As a consequence, iDCs were continuously matured and more CTLs were activated and recruited into the distant tumors. Finally, strong and sustainable immune effects generated abscopal effects, which successfully inhibit lung metastases.

**Figure 6:**
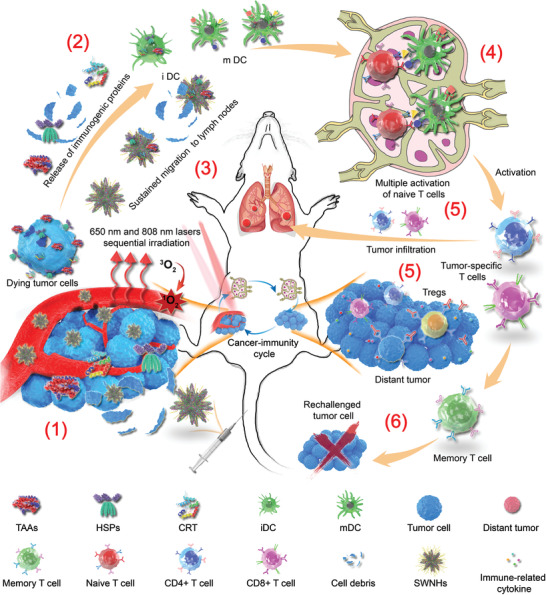
The schematic illustration of synergetic immune responses induced by Gd‐Ce6@SWNHs‐mediated PDT and PTT sequential therapy to eliminate metastatic tumors. This strategy includes the following steps: 1) More diverse immunogenic proteins were released by dying tumor cells after sequential irritations at 650 and 808 nm, respectively. 2) Activation of immature DCs by proteins as mentioned above and cell debris in treated tumors or 3) maturation of DCs by tumor‐associated antigens loaded Gd‐Ce6@SWNHs continuously migrating from the treated tumor to lymph nodes. 4) Activation of tumor‐specific T cells in lymph nodes. 5) Infiltration of CD4+/CD8+ in distant tumors and secretion of immune‐related cytokines of treated tumors in situ. 6) As the results of those synergistic responses, primary tumor, pulmonary metastases, and rechallenged tumor cells could be effectively inhibited or even eliminated via both immune abscopal and immune‐memory effects. Abbreviation representative: TAAs, tumor‐associated antigens; HSPs, heat shock proteins; CRT, calreticulin; iDCs, immature dendritic cells; mDCs, mature dendritic cells; SWNHs, Gd‐Ce6@SWNHs.

In summary, we have engineered a multitask theranostic platform Gd‐Ce6@SWNHs to study the synergistic immunologic responses triggered by sequential PDT and PTT on the primary tumor. Gd‐Ce6@SWNHs have a high tumor targeting efficiency. PDT + PTT in sequence eradicated the primary tumors and generated more diverse DAMPs in situ, eliciting a complementary and synergetic immune response. As a result, durable host antitumor immune effect and significant immune memory effect were elicited after the combination phototherapies to enable effective ablation of pulmonary metastases and protection of mice from tumor cell rechallenge. Therefore, our study has demonstrated the great potency of combined immune‐stimulating therapies of PDT + PTT using Gd‐Ce6@SWNHs and provided a potential way toward tumor synergistic immunotherapy, which is promising for the elimination of tumor metastases and inhibition of recurrence in the clinic.

## Experimental Section

Experimental details are provided in the Supporting Information.

## Conflict of Interest

The authors declare no conflict of interest.

## Supporting information

Supporting Information

Supplemental Video 1

Supplemental Video 2

Supplemental Video 3

Supplemental Video 4

Supplemental Video 5

Supplemental Video 6

Supplemental Video 7

Supplemental Video 8

## References

[1] L. Wan , K. Pantel , Y. Kang , Nat. Med. 2013, 19, 1450. 24202397 10.1038/nm.3391

[2] J. A. Joyce , J. W. Pollard , Nat. Rev. Cancer 2009, 9, 239. 19279573 10.1038/nrc2618 PMC3251309

[3] M. Yarchoan , B. A. Johnson , E. R. Lutz , D. A. Laheru , E. M. Jaffee , Nat. Rev. Cancer 2017, 17, 209. 28233802 10.1038/nrc.2016.154 PMC5575801

[4] S. Gao , D. Yang , Y. Fang , X. Lin , X. Jin , Q. Wang , X. Wang , L. Ke , K. Shi , Theranostics 2019, 9, 126. 30662558 10.7150/thno.29431 PMC6332787

[5] P. Gotwals , S. Cameron , D. Cipolletta , V. Cremasco , A. Crystal , B. Hewes , B. Mueller , S. Quaratino , C. Sabatos‐Peyton , L. Petruzzelli , J. A. Engelman , G. Dranoff , Nat. Rev. Cancer 2017, 17, 286. 28338065 10.1038/nrc.2017.17

[6] J. Nam , S. Son , K. S. Park , W. Zou , L. D. Shea , J. J. Moon , Nat. Rev. Mater. 2019, 4, 398.

[7] Z. Meng , X. Zhou , J. Xu , X. Han , Z. Dong , H. Wang , Y. Zhang , J. She , L. Xu , C. Wang , Z. Liu , Adv. Mater. 2019, 31, 1900927. 10.1002/adma.201900927 31012164

[8] J. Peng , Y. Xiao , W. Li , Q. Yang , L. Tan , Y. Jia , Y. Qu , Z. Qian , Adv. Sci. 2018, 5, 1700891. 10.1002/advs.201700891 PMC5979747 29876215

[9] F. Zhou , J. Yang , Y. Zhang , M. Liu , M. L. Lang , M. Li , W. R. Chen , Clin. Cancer. Res. 2018, 24, 5335. 30068705 10.1158/1078-0432.CCR-18-1126 PMC6214772

[10] W. Li , J. Yang , L. Luo , M. Jiang , B. Qin , H. Yin , C. Zhu , X. Yuan , J. Zhang , Z. Luo , Y. Du , Q. Li , Y. Lou , Y. Qiu , J. You , Nat. Commun. 2019, 10, 3349. 31350406 10.1038/s41467-019-11269-8 PMC6659660

[11] D. Wang , T. Wang , J. Liu , H. Yu , S. Jiao , B. Feng , F. Zhou , Y. Fu , Q. Yin , P. Zhang , Z. Zhang , Z. Zhou , Y. Li , Nano Lett. 2016, 16, 5503. 27525587 10.1021/acs.nanolett.6b01994

[12] Y. Min , K. C. Roche , S. Tian , M. J. Eblan , K. P. McKinnon , J. M. Caster , S. Chai , L. E. Herring , L. Zhang , T. Zhang , J. M. DeSimone , J. E. Tepper , B. G. Vincent , J. S. Serody , A. Z. Wang , Nat. Nanotechnol. 2017, 12, 877. 28650437 10.1038/nnano.2017.113 PMC5587366

[13] C. He , X. Duan , N. Guo , C. Chan , C. Poon , R. R. Weichselbaum , W. Lin , Nat. Commun. 2016, 7, 12499. 27530650 10.1038/ncomms12499 PMC4992065

[14] G. Zhu , L. Mei , H. D. Vishwasrao , O. Jacobson , Z. Wang , Y. Liu , B. C. Yung , X. Fu , A. Jin , G. Niu , Q. Wang , F. Zhang , H. Shroff , X. Chen , Nat. Commun. 2017, 8, 1482. 29133898 10.1038/s41467-017-01386-7 PMC5684198

[15] Y. Liu , Y. Pan , W. Cao , F. Xia , B. Liu , J. Niu , G. Alfranca , X. Sun , L. Ma , J. M. de la Fuente , J. Song , J. Ni , D. Cui , Theranostics 2019, 9, 6867. 31660074 10.7150/thno.37586 PMC6815945

[16] J. Yang , H. Su , W. Sun , J. Cai , S. Liu , Y. Chai , C. Zhang , Theranostics 2018, 8, 1966. 29556368 10.7150/thno.23848 PMC5858512

[17] A. J. Moy , J. W. Tunnell , Adv. Drug Delivery Rev. 2017, 114, 175. 10.1016/j.addr.2017.06.008 28625829

[18] X. S. Li , S. Kolemen , J. Yoon , E. U. Akkaya , Adv. Funct. Mater. 2017, 27, 1604053.

[19] P. Vijayaraghavan , C. H. Liu , R. Vankayala , C. S. Chiang , K. C. Hwang , Adv. Mater. 2014, 26, 6689. 25042520 10.1002/adma.201400703

[20] D. R. Green , T. Ferguson , L. Zitvogel , G. Kroemer , Nat. Rev. Immunol. 2009, 9, 353. 19365408 10.1038/nri2545 PMC2818721

[21] S. Toraya‐Brown , S. Fiering , Int. J. Hyperthermia 2014, 30, 531. 25430985 10.3109/02656736.2014.968640 PMC4558619

[22] S. Gai , G. Yang , P. Yang , F. He , J. Lin , D. Jin , B. Xing , Nano Today 2018, 19, 146.

[23] C. Gao , P. Dong , Z. Lin , X. Guo , B. P. Jiang , S. Ji , H. Liang , X. C. Shen , Chem. ‐ Eur. J. 2018, 24, 12827. 29978545 10.1002/chem.201802611

[24] Y. Ma , Y. Zhang , X. Li , Y. Zhao , M. Li , W. Jiang , X. Tang , J. Dou , L. Lu , F. Wang , Y. Wang , ACS Nano 2019, 13, 11967. 31553168 10.1021/acsnano.9b06040

[25] C. Wang , L. Xu , C. Liang , J. Xiang , R. Peng , Z. Liu , Adv. Mater. 2014, 26, 8154. 25331930 10.1002/adma.201402996

[26] Y. Cao , Y. Ma , M. Zhang , H. Wang , X. Tu , H. Shen , J. Dai , H. Guo , Z. Zhang , Adv. Funct. Mater. 2014, 24, 6963.

[27] S. Iijima , M. Yudasaka , R. Yamada , S. Bandow , K. Suenaga , F. Kokai , K. Takahashi , Chem. Phys. Lett. 1999, 309, 165.

[28] N. Karousis , I. Suarez‐Martinez , C. P. Ewels , N. Tagmatarchis , Chem. Rev. 2016, 116, 4850. 27074223 10.1021/acs.chemrev.5b00611

[29] B. He , Y. Shi , Y. Liang , A. Yang , Z. Fan , L. Yuan , X. Zou , X. Chang , H. Zhang , X. Wang , W. Dai , Y. Wang , Q. Zhang , Nat. Commun. 2018, 9, 2393. 29921862 10.1038/s41467-018-04700-z PMC6008334

[30] J. Miyawaki , M. Yudasaka , T. Azami , Y. Kubo , S. Iijima , ACS Nano 2008, 2, 213. 19206621 10.1021/nn700185t

[31] K. Welsher , Z. Liu , S. P. Sherlock , J. T. Robinson , Z. Chen , D. Daranciang , H. Dai , Nat. Nanotechnol. 2009, 4, 773. 19893526 10.1038/nnano.2009.294 PMC2834239

[32] Z. Liu , A. C. Fan , K. Rakhra , S. Sherlock , A. Goodwin , X. Chen , Q. Yang , D. W. Felsher , H. Dai , Angew. Chem. 2009, 121, 7804. 10.1002/anie.200902612 PMC2824548 19760685

[33] X. Song , C. Liang , H. Gong , Q. Chen , C. Wang , Z. Liu , Small 2015, 11, 3932. 25925790 10.1002/smll.201500550

[34] D. Wang , L. Meng , Z. Fei , C. Hou , J. Long , L. Zeng , P. J. Dyson , P. Huang , Nanoscale 2018, 10, 8536. 29694478 10.1039/c8nr00663f

[35] D. Meng , S. Yang , L. Guo , G. Li , J. Ge , Y. Huang , C. W. Bielawski , J. Geng , Chem. Commun. 2014, 50, 14345. 10.1039/c4cc06849a 25286834

[36] J. T. Robinson , S. M. Tabakman , Y. Liang , H. Wang , H. S. Casalongue , D. Vinh , H. Dai , J. Am. Chem. Soc. 2011, 133, 6825. 21476500 10.1021/ja2010175

[37] L. Zhang , H. Su , J. Cai , D. Cheng , Y. Ma , J. Zhang , C. Zhou , S. Liu , H. Shi , Y. Zhang , C. Zhang , ACS Nano 2016, 10, 10404. 27934087 10.1021/acsnano.6b06267

[38] H. Zhang , H. Wu , J. Wang , Y. Yang , D. Wu , Y. Zhang , Y. Zhang , Z. Zhou , S. Yang , Biomaterials 2015, 42, 66. 25542794 10.1016/j.biomaterials.2014.11.055

[39] C. Yue , Y. Yang , C. Zhang , G. Alfranca , S. Cheng , L. Ma , Y. Liu , X. Zhi , J. Ni , W. Jiang , J. Song , J. M. de la Fuente , D. Cui , Theranostics 2016, 6, 2352. 27877240 10.7150/thno.15433 PMC5118600

[40] C. Luo , B. Sun , C. Wang , X. Zhang , Y. Chen , Q. Chen , H. Yu , H. Zhao , M. Sun , Z. Li , H. Zhang , Q. Kan , Y. Wang , Z. He , J. Sun , J. Controlled Release 2019, 302, 79. 10.1016/j.jconrel.2019.04.001 30946853

[41] H. Wu , H. Jin , C. Wang , Z. Zhang , H. Ruan , L. Sun , C. Yang , Y. Li , W. Qin , C. Wang , ACS Appl. Mater. Interfaces 2017, 9, 9426. 28247750 10.1021/acsami.6b16844

[42] K. Hayashi , M. Nakamura , H. Miki , S. Ozaki , M. Abe , T. Matsumoto , T. Kori , K. Ishimura , Adv. Funct. Mater. 2014, 24, 503.

[43] X. Duan , C. Chan , N. Guo , W. Han , R. R. Weichselbaum , W. Lin , J. Am. Chem. Soc. 2016, 138, 16686. 27976881 10.1021/jacs.6b09538 PMC5667903

[44] S. Rose‐John , K. Winthrop , L. Calabrese , Nat. Rev. Rheumatol. 2017, 13, 399. 28615731 10.1038/nrrheum.2017.83

[45] S. L. Topalian , J. M. Taube , R. A. Anders , D. M. Pardoll , Nat. Rev. Cancer 2016, 16, 275. 27079802 10.1038/nrc.2016.36 PMC5381938

[46] D. Kalyane , N. Raval , R. Maheshwari , V. Tambe , K. Kalia , R. K. Tekade , Mater. Sci. Eng., C 2019, 98, 1252. 10.1016/j.msec.2019.01.066 30813007

[47] H. Kobayashi , R. Watanabe , P. L. Choyke , Theranostics 2013, 4, 81. 24396516 10.7150/thno.7193 PMC3881228

[48] Z. Liu , W. Cai , L. He , N. Nakayama , K. Chen , X. Sun , X. Chen , H. Dai , Nat. Nanotechnol. 2007, 2, 47. 18654207 10.1038/nnano.2006.170

[49] S. Xue , C. Zhang , Y. Yang , L. Zhang , D. Cheng , J. Zhang , H. Shi , Y. Zhang , J. Biomed. Nanotechnol. 2015, 11, 1027. 26353592 10.1166/jbn.2015.2023

[50] L. Zhang , H. Su , H. Wang , Q. Li , X. Li , C. Zhou , J. Xu , Y. Chai , X. Liang , L. Xiong , C. Zhang , Theranostics 2019, 9, 1893. 31037146 10.7150/thno.30523 PMC6485290

[51] M. Yu , J. Zheng , ACS Nano 2015, 9, 6655. 26149184 10.1021/acsnano.5b01320 PMC4955575

[52] X. Liang , X. Y. Ye , C. Wang , C. Y. Xing , Q. W. Miao , Z. J. Xie , X. L. Chen , X. D. Zhang , H. Zhang , L. Mei , J. Controlled Release 2019, 296, 150. 10.1016/j.jconrel.2019.01.027 30682441

[53] S. Nourshargh , R. Alon , Immunity 2014, 41, 694. 25517612 10.1016/j.immuni.2014.10.008

[54] T. N. Schumacher , R. D. Schreiber , Science 2015, 348, 69. 25838375 10.1126/science.aaa4971

[55] D. S. Chen , I. Mellman , Immunity 2013, 39, 1. 23890059 10.1016/j.immuni.2013.07.012

