# Supplementary material for: Sequential PDT and PTT Using Dual‐Modal Single‐Walled Carbon Nanohorns Synergistically Promote Systemic Immune Responses against Tumor Metastasis and Relapse
Source: Adv Sci (Weinh). 2020 Jul 1;7(16):2001088. doi: 10.1002/advs.202001088 (PMC7435231; doi:10.1002/advs.202001088)
Supplement: Supplementary file 1 — Supporting Information [file ADVS-7-2001088-s001.pdf]

**Supporting information**

# Sequential PDT and PTT Using Dual-modal Single-walled Carbon Nanohorns Synergistically Promote Systemic Immune Responses Against Tumor Metastases

*Jingxing Yang, Mengfei Hou, Wenshe Sun, Qinghe Wu, Jia Xu, Liqin Xiong, Yimin Chai, Yuxin Liu, Meihua Yu, Haolu Wang, Zhi Ping Xu, Xiaowen Liang*, Chunfu Zhang **

Dr J. Yang, Dr J. Xu, Dr Y Chai, Prof C. Zhang
Department of Orthopedics, Shanghai Jiao Tong University Affiliated 6th Hospital, School of Biomedical Engineering, Shanghai Jiao Tong University, Shanghai 200030, China.

Dr J. Yang, Dr M. Hou, Dr W. Sun, Dr Q. Wu, Dr L. Xiong, Prof C. Zhang
Department of Nuclear Medicine, Rui Jin Hospital, School of Biomedical Engineering, Shanghai Jiao Tong University, Shanghai 200030, China.

Ms Y. Liu
School of Environment and Biological Engineering, Nanjing University of Science and Technology, Nanjing, Jiangsu 210094, China.

Dr M. Yu, Dr H. Wang, Dr X. Liang
The University of Queensland Diamantina Institute, The University of Queensland, Woolloongabba, Queensland 4102, Australia.

Dr M. Yu, Prof Z.P.Xu
Australian Institute for Bioengineering and Nanotechnology, The University of Queensland, St Lucia, Brisbane, QLD 4072, Australia

Dr H. Wang, Dr X. Liang
Gallipoli Medical Research Institute, Greenslopes Private Hospital, Greenslopes, Queensland 4120, Australia.

Dr H. Wang
Department of Biliary-Pancreatic Surgery, Ren Ji Hospital, School of Medicine, Shanghai Jiao Tong University, 800, Dongchuan Road, Shanghai 200240, China.

Dr X. Liang
Department of General Surgery, Changzheng Hospital, The Second Military Medical University, Shanghai, China.

* Corresponding authors: Prof Chunfu Zhang, Tel: 0086-21-62933323; Fax: 0086-21-62933323; E-mail: cfzhang@sjtu.edu.cn; Dr Xiaowen Liang, E-mail: x.liang@uq.edu.au

## Experimental Section

**Cell lines.** The murine breast cancer 4T1 cell lines were acquired from the American Type Culture Collection (ATCC), and firefly luciferase-stable-transfected 4T1 (fLuc-4T1) was prepared, screened, and identified in our laboratory.[1] The cells were cultured in RPMI 1640 supplemented with 10% fetal bovine serum, 100 μg $mL^{-1}$ streptomycin and 100 U $mL^{-1}$ penicillin, and in 5% $CO_2$ at 37°C for candidate culture conditions. Dendritic cells were isolated from the bone marrow of 8-week-age Balb/c mice (18-20 g), which were purchased from Shanghai Slack Laboratory Animal Co., Ltd. (Slack, Shanghai, China). All products of cultured cells were purchased from the Gibco company (Invitrogen, Carlsbad, CA, USA).

**Animal.** Balb/c mice (18-20 g) were obtained from Shanghai Slack Laboratory Animal Co., Ltd. Female mice between 6-week and 10-week old were used. All animal procedures were carried out in accordance with the guidelines approved by the Institutional Animal Care and Use Committee (IACUC) of the Shanghai Jiao Tong University.

**Materials.** Pristine SWNHs was purchased from Sigma-Aldrich (Merck, NJ, USA). Poly (lactide-co-glycolide)-b-Poly(ethylene glycol)-maleimide copolymers (PLGA20k-PEG5k-Mal, Mw ~20,000-5,000 Da, 50:50 LA:GA) was obtained from Polyscitech (Cat: AI020, Akina, Inc., West Lafayette, OH, USA). The Live/Dead Zombie Aqua™ Fixable Viability Kit (Cat: 423102) and all the fluorescence-labeled antibodies were obtained from BioLegend (BioLegend, Inc., San Diego, CA). The related peptides applied in the qPCR analysis were obtained from Sangon (Sangon Biotech, Inc., Shanghai, China), and the regents involved in the analysis of gene expression were obtained from BD (Becton, Dickinson and Company, Franklin Lake, NJ). The specific category of product-related would be mentioned in the corresponding experimental methods. Poly (maleic anhydride-alt-1-octadecene) ($C_{18}$PMH, Mw 1051 Da), tetrahydrofuran (THF) and the other chemicals were obtained from Sigma-Aldrich (Merck KGaA, Darmstadt, Germany) unless otherwise noted.

**Synthesis and Characterization of Gd-Ce6@SWNHs.** Gd-Ce6@SWNHs was synthesized according to our previous report.[2] Briefly, to modify pristine SWNHs, PEG-PLGA (10 mg) and $C_{18}$PMH (10 mg) were mixed into 1 mL of THF and then added dropwise into 1 mL of aqueous SWNHs suspension (1 mg mL$^{-1}$). The mixture was sonicated at a cold water-bath with nitrogen bubbling for half an hour to evaporate THF. And then kept sonicating for another 1-2 hours to yield a black suspension. The suspension was centrifuged at 10,000 rpm for 30 min to remove any large SWNHs aggregates. Then the dual-polymer-coated SWNHs were collected by ultrafiltration (Amicon Ultra-4, MWCO 100 K, Merck Millipore, Darmstadt, Germany).

To load Ce6, 1 mL of Ce6 aqueous solutions with various concentrations (0.5-6 mg mL$^{-1}$) was added into 1 mL of dual polymer-coated SWNHs suspension (1 mg mL$^{-1}$). The mixture was stirred for 1 h at room temperature and then sonicated for an additional 1 h. The excess Ce6 was removed by dialysis (Mw~8,000-14,000) against deionized water overnight. The drug-loading capacity (DLC) and drug-loading efficiency (DLE) were evaluated by the fluorescence signal of Ce6@SWNHs dialysate against known standards spectrum of free Ce6 (Ex=408 nm/Em=650 nm).

$Gd^{3+}$ labeling was achieved through chelating Gd ions with porphyrin structures in Ce6.[3-4] The final product, termed Gd-Ce6@SWNHs, was purified by dialysis (Millipore, MWCO 3 k) against deionized water, retrieved by ultrafiltration (MWCO 100 K) and then freeze-dried.

The hydrodynamic sizes and zeta potentials were examined by dynamic light scattering (DLS, Malvern Zetasizer Nano ZSP, Malvern, United Kingdom) in deionized water at 25°C. Transmission electron microscopy (TEM) images were obtained from JEM-2011 (JEOL, Japan) at 200 kV. NIR absorption spectra were recorded with a NanoDrop 1000 (Thermo Scientific, Wilmington, USA). Excitation and emission spectra of nanosystems were evaluated by a fluorescence spectrophotometer (F-2700, HITACHI, Tokyo, Japan), and the BET specific surface area was detected by automated gas sorption analyzer (Autosorb, iQ2, Quantachrome

Instruments, FL, USA). The concentrations of gadolinium were measured by ICP-MS (Z-2000 series, HITACHI, Tokyo, Japan) and then T1 relaxivity of Gd-Ce6@SWNHs was determined by linear fitting the reverse T1 relaxation times measured by an NMR analyzer (Mini spec mq60, Bruker, Karlsruhe, Germany) as a function of $Gd^{3+}$ concentrations (in mM).

**Phototherapy using Gd-Ce6@SWNHs *in vitro*.** To assess the photothermal performance of Gd-Ce6@SWNHs, the lyophilized nanoparticles were resuspended into 400 µL of deionized water at different concentrations (0, 5, 10, 20, and 50 µg $mL^{-1}$ in SWNHs) and then illuminated with an 808 nm laser at a power intensity of 1 $Wcm^{-2}$ or irradiated at different power densities (0.5, 1, 1.5 and 2 W $cm^{-2}$). A digital thermometer was used to monitor temperature change with a thermocouple probe submerged in the solution. To test the thermal stability, Gd-Ce6@SWNHs (20 µg $mL^{-1}$) was alternatingly irradiated for six times at the power density of 1 W $cm^{-2}$ for 5 min. The digital infrared-thermal photos were captured by IR thermal camera (FOTRIC, Shanghai, China). For analysis of the photothermal conversion efficiency (η) under 808 nm laser irradiation, correlation equations were applied in the above-recorded data according to the previous report.[5-6]

$$\eta = \frac{hS\Delta T_{max} - Q_s}{I(1-10^{-A})} = \frac{hS(\Delta T_{max} - \Delta T_{max_S})}{I(1-10^{-A})} \quad (1)$$

$$hS = \frac{m_s C_s}{\tau} \quad (2)$$

Where $h$ is the transfer coefficient, $S$ is the surface area of the container, $\Delta T_{max}$ represents the temperature change of sample at the maximum steady-state temperature; $\Delta T_{max_S}$ represents the temperature change of surroundings; $Q_s$ represents the heat dissipation from the light absorbed by the quartz sample cell; $I$ is the incident NIR laser (808 nm) power; $A$ is the absorbance of the nanoparticles at 808 nm; $m_s$ and $C_s$ are the mass and heat capacity of deionized water, respectively; $\tau$ is the sample system time constant, which is determined by the linear curve fitting of temperature cooling time *vs* its $\ln\frac{\Delta T}{\Delta T_{max}}$.

To evaluate the potential synergistic phototherapy of Gd-Ce6@SWNHs on

tumor cells, 4T1 cells (5000/well) were first seeded into 96-well plates, grow to ~60%-70% confluence and then incubated with various concentrations of Gd-Ce6@SWNHs nano-vehicles (1, 3, 5, 10, 20, and 50 μg $mL^{-1}$ in SWNHs) for 12 h. Then the culture media were removed and cells were washed with PBS three times and supplemented with fresh media (RPMI-1640, 200 μL/well). For PDT, the cells were illuminated by a 650 nm laser for different periods of time (3, 5, 10, and 15 min) at a density of 40 mW $cm^{-2}$. For PTT, the cells were irradiated by an 808 nm laser for 5 min at different power densities (1, 1.5 and 2 W $cm^{-2}$). For PDT + PTT combination therapy, the cells were exposed to the 650 nm laser at the power density of 40 mW $cm^{-2}$ and the 808 lasers at 1.5 W $cm^{-2}$ for 5 min sequentially. After the treatments, cells were supplemented with fresh media and further maintained for an additional 24 h. Finally, the cell viability was evaluated by CCK-8 assay as described previously.[7-8] Accordingly, the half-maximal inhibitory concentration values ($IC_{50}$) of PDT, PTT, or PDT + PTT combination therapy were determined from dose-response curves. The combination index (CI), which indicates the type of anticancer mechanism of the combined therapy, was calculated according to the formula $\mathrm{CI} = \frac{C1}{CX1} + \frac{C2}{CX2} \quad (3)$

Where $C1$ and $C2$ are the concentrations of the first and the second therapeutic required to achieve a certain effect in combination therapy, and $CX1$ and $CX2$ are the concentrations of the first and the second drugs that generate an identical effect alone. When $CI$ < 1, = 1, or > 1, the two therapeutics are implied to have synergistic, additive, or antagonistic effects, respectively.[9-11]

The therapeutic effects of PTT and PDT were also evaluated by immunohistological chemistry. For PTT, 4T1 cells were co-incubated with Gd-Ce6@SWNHs (10 μg $mL^{-1}$) for 12 h and irradiated with an 808 nm laser (1.5 W $cm^{-2}$) for 3, 5, and 7 min. Live-dead cell staining was performed by Calcein-AM/PI staining kit (Cat: 40747ES76, Yeasen Biotech Co., Ltd., Shanghai, China). Namely, the live cells could be penetrated by the green fluorescent dye (Ex/Em = 488/518 nm). However, since the dead cells could be simply stained with pyridinium iodide (PI), a red fluorescent dye which could hardly penetrate the live cell membrane (Ex/Em =

488/615 nm). After staining, the cells were examined using a dark-field fluorescence microscope ECLIPSE Ti-E (Nikon, Tokyo, Japan). Singlet oxygen generation after PDT was determined by the DCFH-DA assay.[12-13] The DCFH-DA is sensitive to SO and can be oxidized to a strong green fluorescent substance, dichlorofluorescein (DCF). So reactive oxygen species could be detected through DCFH-DA assay kit (Ex/Em = 488/525 nm, Beyotime, Shanghai, China). For CLSM (TCS SP8 STED 3X, Leica, Brunswick, Germany) imaging, 4T1 cells were seeded on 10 $mm^2$ glass coverslips placed in six-well plates at a density of $5\times10^4$ cells per well and incubated with Gd-Ce6@SWNHs at a concentration of 10 μg $mL^{-1}$ (in SWNHs) for 12 h. After incubation, the cells were irradiated by a 650 nm laser at a light dose of 40 mW $cm^{-2}$ for 3, 5, 7 and 10 min, stained by DCFH-DA assay kit and examined by CLSM.

**Phototherapy-induced immune response *in vitro*.** The ICD induced by PTT and PDT were evaluated by immunohistological staining against heat shock proteins 70 (HSP 70) and calreticulin (CRT), respectively. 4T1 cells were seeded on cover slides in six-well plates at $5\times10^5$ cells per well and cultured for 24 h. Then the culture media were replaced with fresh media containing 10 μg $mL^{-1}$ of Gd-Ce6@SWNHs and the cells were incubated for 12 h. Subsequently, the media were replaced by the fresh one and the cells were irradiated by a 650 nm laser at the density of 40 mW $cm^{-2}$ for 5 min. After the treatment, the cells were fixed with 4% formaldehyde for 15 minutes and rinsed with PBS (pH = 7.4) three times. For CRT staining, the cells were incubated with primary calreticulin monoclonal antibody (Cell Signaling Technology, Boston, USA) overnight at 4°C, followed by the Alexa Fluor 488-conjugated anti-secondary antibody (Cell Signaling Technology, Boston, USA) for 1 hour at room temperature in the dark. The cell nuclei were counter-stained with DAPI. For heat shock protein staining in the PTT group, the Gd-Ce6@SWNHs-treated cells were illuminated by an 808 nm laser at 1.5 W $cm^{-2}$ for 5 min and stained with primary HSP70 monoclonal antibody (Leading Biology, California, USA) and Alexa Fluor 488-conjugated anti-secondary antibody (Leading Biology, California, USA). The stained cells were examined by CLSM.

To further demonstrate the immunogenicity induced by phototherapy, activation of DCs was evaluated using a transwell system. The immature DCs (iDCs) were first derived from the bone marrow of 8-week-old Blab/c mice according to the previous reports.[14] The iDCs were incubated with Gd-Ce6@SWNHs or PEGylated SWNHs at different concentrations (0-20 μg $mL^{-1}$) for 12 h to examine the effect of the probe on DCs maturation. To assess PDT and PTT induced immunogenicity, 4T1 cells were co-cultured with Gd-Ce6@SWNHs (10 μg $mL^{-1}$) for 12 h, and then transferred to upper chamber of a transwell system and illuminated by a 650 nm laser (40 mW $cm^{-2}$) (PDT) or 808 nm lasers (1.5 W $cm^{-2}$) (PTT) for 5 min. DCs were cocultured in the lower chamber (Supporting Figure S9). DCs activation was examined by flow cytometry and the related gene expression was analyzed by RT-PCR. For flow cytometry assay, DCs maturation markers were stained with anti-CD11c-APC (Clone: N418), anti-CD80-PE/Cy7 (Clone: 16-10A1), anti-CD86-PE (Clone: GL-1) antibodies, and then analyzed by flow cytometry (BD FACSAria Ⅱ, NJ) according to manufacturer′s instructions. For gene expression analysis, DCs were collected and the total RNA was extracted using the RNeasy RNA Isolation Kit (Cat: AM1912, Invitrogen, CA). The RNA was reverse transcribed (Applied Biosystems, CA) and various proteins′ mRNA were detected by RT-qPCR system (7900HT, Applied Biosystems, CA) using an SYBR Green assay kit (Invitrogen, CA). The values obtained for the threshold cycle for each gene were normalized using the average of housekeeping genes GAPDH amplified on the same cycle. The primer sequences for RT-qPCR were as follows:

| | | |
|---|---|---|
| IFN-γ | sense | 5′-TTACTGCCACGGCACAGTCA-3′ |
| | antisense | 5′-AGTTCCTCCAGATATCCAAGAAGAGA-3′ |
| TNF-α | sense | 5′-TCTTCTCGAACCCCGAGTGA-3′ |
| | antisense | 5′-CCTCTGATGGCACCACCAG-3′ |
| CD80 | sense | 5′-TGCTGTCTGTCATTGCTGGGAAACT-3′ |
| | antisense | 5′-CCCAGGTGAAGTCCTCTGACACGTG-3′ |
| CD86 | sense | 5′-TCCAGAACTTACGGAAGCACCCACG-3′ |

| | | |
|---|---|---|
| | antisense | 5′-CAGGTTCACTGAAGTTGGCGATCAC-3′ |
| IL-1β | sense | 5′-ACAGACCTTCCAGGAGAATG-3′ |
| | antisense | 5′-GCAGTTCAGTGATCGTACAG-3′ |
| IL-10 | sense | 5′-GTGATGCCCCAAGCTGAGA-3′ |
| | antisense | 5′- CACGGCCTTGCTCTTGTTTT-3′ |
| IL-12p35 | sense | 5′-TGGAGTGCCAGGAGGACAGT-3′ |
| | antisense | 5′- TCTTGGGTGGGTCAGGTTTG-3′ |

***In vivo* imaging.** Female mice with 6-10 weeks old were used for preparing the orthotopic breast cancer model by implanting 4T1 cells ($1\times10^6$) into the breast fat pad. When tumors grew around 100 $mm^3$, tumor-bearing mice were i.v. administered with Gd-Ce6@SWNHs at the dose of 10 mg $kg^{-1}$ b.w. (in SWNHs). Then the mice were imaged at determined time points (1, 3, 6, 12 and 24 h). Specifically, fluorescence imaging was performed using the IVIS Lumina optical imaging system (PerkinElmer, MA, UAS). T1-weighted MR imaging was conducted using a 7.0-T clinical MRI scanner (Bruker ClinScan, MA) with a special coil for small animal imaging. MSOT and PA imaging were performed using MSOT inVision 256 (iThera Medical, Germany) under NIR-Ⅰ region (600-900 nm) and VisualSonics/VEVO LAZR-X (Fujifilm, Tokyo, Japan) at a NIR-Ⅱ region (1100-1900 nm), respectively. Three-dimensional visualization can be used to render three-dimensional volumes of mice using a set of two-dimensional images, which integrated into ViewMSOT software.

**Pharmacokinetics and biodistributions.** 4T1 tumor-bearing mice were i.v. injected with Gd-Ce6@SWNHs at the dose of 10 mg $kg^{-1}$ b.w. and MSOT imaging was performed and the signal intensity of the jugular vein was monitored in real-time. The blood-half life of Gd-Ce6@SWNHs was determined by fitting PA signals as a function of post-injection time according to the two-compartment model.[15-16] MSOT imaging of major organs (e.g. livers, spleens, lungs, kidneys, intestine) and tumors were also recorded. The biodistribution of Gd-Ce6@SWNHs and its continuous migration from the tumor site to tumor-draining lymph nodes were examined by

MSOT 3D reconstruction *in vivo* and FI of the isolated organs *ex vivo*.

**PDT and PTT combination therapy on tumors.** To establish 4T1 orthotopic murine breast cancer model with spontaneous lung metastasis, 4T1 cells ($1\times10^6$) were first implanted into the breast fat pad. After implantation for 10-14 days, tumor lung metastases occurred and were determined by H&E staining of lung slice in five mice randomly. When the volume of the primary tumors reached 80-100 $mm^3$, the mice were divided into five groups with ten mice in each group: PBS, Gd-Ce6@SWNHs, PDT, PTT, and PDT+PTT group. Gd-Ce6@SWNHs was administered at the dosage of 10 mg $kg^{-1}$ b.w. For PDT therapy, the mice were exposed to a 650 nm laser for 10 min with an intensity of 40 mW $cm^{-2}$. For PTT, the tumors were irradiated with an 808 nm laser at the power intensity of 0.5 W $cm^{-2}$ for 10 min. Temperature changes of tumors were monitored with an InfraRed (IR) camera (FOTRIC Systems, Shanghai, China). PDT + PTT combination therapy was performed by treating the tumors with PDT and PTT sequentially. After the treatments, the size of the tumor was measured by micrometer caliper and tumor volume was calculated according to the following formulation:

$$V(mm^3) = width^2 \times \frac{length}{2} \quad (4)$$

**Abscopal therapeutic effect on the distant tumor.** To elucidate the mechanism of the therapeutic effect of the combination therapy on pulmonary metastases, the immune abscopal effect possibly induced by the phototherapies was next assessed using the bilateral tumor model. The bilateral tumor model was developed by implanting 4T1 cells ($1\times10^6$) into the left breast fat pad as the primary tumor and into the right one as the distant tumors at 5-day-post the first implantation. After maintained for another 5 days, the mice were i.v. injected with Gd-Ce6@SWNHs at the dose of 10 mg $kg^{-1}$ b.w. Twenty four hours after injection, the primary tumors were irradiated by 650 nm (PDT) and 808 nm (PTT) lasers sequentially with the same conditions described previously. Subsequently, the sizes of the primary and distant tumors and the survival of mice were monitored within 70 days.

For immunoassay, sera, distant tumors, and TDLNs were collected at day 3, 7 and

14 post-irradiation. The mouse sera were collected by using orbital sinus blood sampling and the immune factors (i.e. TNF-α, IFN-γ, IL-α, IL-1β, IL-2, IL-6, IL-10, IL-12, and G-CSF) were measured by Luminex liquid suspension chip technology (Bio-Plex MAGPIX System, Luminex, Texas, USA).

CTL cells infiltration into the distant tumors and DCs in both tumor-draining lymph node and the distant tumors were evaluated using the flow cytometry assay. For this purpose, the distant tumors and TDLNs were harvested at 3, 7 and 14 days after the treatments, processed through mechanical disruption and digested for 30 min at 37°C in RPMI containing 0.1 mg $mL^{-1}$ DNAse and 0.5 mg $mL^{-1}$ collagenase. The cell suspensions were filtered through a 70 μm cell filter and washed in cold PBS, and then incubated with Fc Block for 15 min on ice before the surface staining. The T cells were stained with anti-CD3-APC (Biolegend, Clone: 17A2), anti-CD45-APC/Fire™750 (Biolegend, Clone: 30-F11), anti-CD8a-PerCP/Cyanine5.5 (Biolegend, Clone: 53-6.7), anti-CD4-FITC (Biolegend, Clone: RM4-5) antibodies according to the manufacturer′s protocols. For analysis of mature of DC cells, the samples were stained with anti-CD45-APC/Fire™750 (Biolegend, Clone: 30-F11), anti-mouse I-A/I-E-PerCP/Cyanine5.5 (Biolegend, Clone: M5/114.15.2), anti-CD11c-APC (Bioscience, Clone: N418), anti-CD80-PE/Cy7 (Bioscience, Clone: 16-10A1), anti-CD86-PE (Bioscience, Clone: GL-1) antibodies. The mature DCs were defined as the co-expression of CD80 and CD86. All samples washed three times with wash buffer before applying flow cytometric analysis. Tumor-infiltrating toxicity T lymphocytes were gated on Live CD45+CD3+CD8+/CD4+ and mDCs were gated on Live CD45+CD11c+I-A/I-E+CD80+CD86+.

**Histopathological study.** For immunofluorescence staining, tumors were collected, frozen and then sectioned into 6 μm thickness slides using a cryostat (UC6-FC6, Leica, Brunswick, Germany). The tissue slides were fixed in cold acetone for 10 min, air-dried and then soaked in PBS (pH = 7.4) for 5 min. After blocking with 20% donkey serum, the slides were incubated with primary anti-mouse monoclonal

antibodies against CD4 (dilution 1:100, eBioscience, CA) and CD8 (dilution 1:50, eBioscience, CA) overnight at 4 ℃ and followed by incubation with fluorescence-conjugated secondary antibodies for 1 h at room temperature. After counterstained with DAPI for another 10 min, the sections were then washed twice with PBS. Photomicrography was performed using a light microscope (Nikon Tokyo, Japan).

For hematoxylin and eosin (H&E) examination, the lung, major organs, and tumors were harvested at the predetermined time and fixed in 4% neutral buffered formalin, embedded in paraffin and processed for H&E (Aladdin, Ontario, CA) staining according to the protocol provided by the manufacturer and previous reports.[8, 17]

**Detection of lung metastases.** The lungs of mice were injected with India ink through the trachea and fixed with Fekete′s solution according to the previous report.[2, 18] For histological examination of the metastases, the lungs were dissected, embedded in paraffin and processed for H&E staining. Tumor metastasis sites subsequently appeared as white nodules in the digital photograph or as the bulk of aggregates in H&E slices. The nodules were counted under a digital microscope.

**Evaluation of immune memory and long-term biocompatibility of Gd-Ce6@SWNHs.** To evaluate the immune memory effect induced by PDT + PTT combination therapy, the cured mice were rechallenged by 4T1 cells with stably expressing the firefly luciferase gene. The fLuc-4T1 cells ($2\times10^5$) were intravenously administered *via* tail vein into the cured mice. The development of neoplasm metastases in the lung was monitored using bioluminescence imaging (IVIS Lumina LT, MA) with D-luciferin, Sodium Salt (Cat: E1605, Promega, WI) as the substrate (150 mg $kg^{-1}$). Mice injected with PBS (pH = 7.4) or subjected to surgical removal of the primary tumor were used as controls. The lung was also examined histologically by H&E staining once the mice died in the control groups during maintenance or were euthanized in the treated group.

To examine the biological safety of Gd-Ce6@SWNHs, major organs and whole blood from the survived mice were carefully examined at 6, 9, and 18 months after

fLuc-4T1 rechallenge. The organs were examined by H&E staining. Hematology and blood biochemistry (BUN, GOT, GPT, WBC, RBC, HGB, HCT, MCV, MCH, and PLT) were analyzed using SYSMEX dry biochemical analysis system (SYSMEX FDC 7000i, Kobe, Japan).

**Statistics** All results were reported as means with standard deviation (SD). For multiple comparisons, a one-way analysis of variance (ANOVA) with Tukey′s multiple comparison tests was used. *P* values of less than 0.05 were considered significant. For the analysis of blood half-life of the nanomaterials *in vivo*, the GraphPad Prism software version 7.0 (GraphPad Software Inc., CA) was performed. The analyses of flow cytometry data were applied in FlowJo software version 10.0 (BD, NJ).

## References


[1] T. Hiraga, P. J. Williams, A. Ueda, D. Tamura, T. Yoneda, *Clin. Cancer Res.* **2004**, *10*, 4559.

[2] J. Yang, H. Su, W. Sun, J. Cai, S. Liu, Y. Chai, C. Zhang, *Theranostics* **2018**, *8*, 1966.

[3] X. Song, C. Liang, H. Gong, Q. Chen, C. Wang, Z. Liu, *Small* **2015**, *11*, 3932.

[4] M. Gao, F. Fan, D. Li, Y. Yu, K. Mao, T. Sun, H. Qian, W. Tao, X. Yang, *Biomaterials* **2017**, *133*, 165.

[5] D. K. Roper, W. Ahn, M. Hoepfner, *J. Phys. Chem. C* **2007**, *111*, 3636.

[6] L. Zhang, H. Su, J. Cai, D. Cheng, Y. Ma, J. Zhang, C. Zhou, S. Liu, H. Shi, Y. Zhang, C. Zhang, *ACS Nano* **2016**, *10*, 10404.

[7] C. Yang, X. Mi, H. Su, J. Yang, Y. Gu, L. Zhang, W. Sun, X. Liang, C. Zhang, *Biomater. Sci.* **2019**, *7*, 2076.

[8] Y. Yang, L. Zhang, J. Cai, X. Li, D. Cheng, H. Su, J. Zhang, S. Liu, H. Shi, Y. Zhang, C. Zhang, *ACS Appl. Mater. Interfaces* **2016**, *8*, 1718.

[9] T. C. Chou, *Cancer Res.* **2010**, *70*, 440.

[10] Y. Li, G. Liu, J. Ma, J. Lin, H. Lin, G. Su, D. Chen, S. Ye, X. Chen, X. Zhu, Z. Hou, *J. Control. Release* **2017**, *258*, 95.

[11] H. Wu, H. Jin, C. Wang, Z. Zhang, H. Ruan, L. Sun, C. Yang, Y. Li, W. Qin, C. Wang, *ACS Appl. Mater. Interfaces* **2017**, *9*, 9426.

[12] D. Wang, T. Wang, J. Liu, H. Yu, S. Jiao, B. Feng, F. Zhou, Y. Fu, Q. Yin, P. Zhang, Z. Zhang, Z. Zhou, Y. Li, *Nano Lett.* **2016**, *16*, 5503.

[13] X. Duan, C. Chan, N. Guo, W. Han, R. R. Weichselbaum, W. Lin, *J. Am. Chem. Soc.* **2016**, *138*, 16686.

[14] C. Wang, L. Xu, C. Liang, J. Xiang, R. Peng, Z. Liu, *Adv. Mater.* **2014**, *26*, 8154.

[15] M. Yu, J. Zheng, *ACS Nano* **2015**, *9*, 6655.

[16] L. Zhang, H. Su, H. Wang, Q. Li, X. Li, C. Zhou, J. Xu, Y. Chai, X. Liang, L. Xiong, C. Zhang, *Theranostics* **2019**, *9*, 1893.

[17] Y. Tang, C. Zhang, J. Wang, X. Lin, L. Zhang, Y. Yang, Y. Wang, Z. Zhang, J. W. Bulte, G. Y. Yang, *Adv. Funct. Mater.* **2015**, *25*, 1024.

[18] C. Liang, S. Diao, C. Wang, H. Gong, T. Liu, G. Hong, X. Shi, H. Dai, Z. Liu, *Adv. Mater.* **2014**, *26*, 5646.

# Supporting Figures

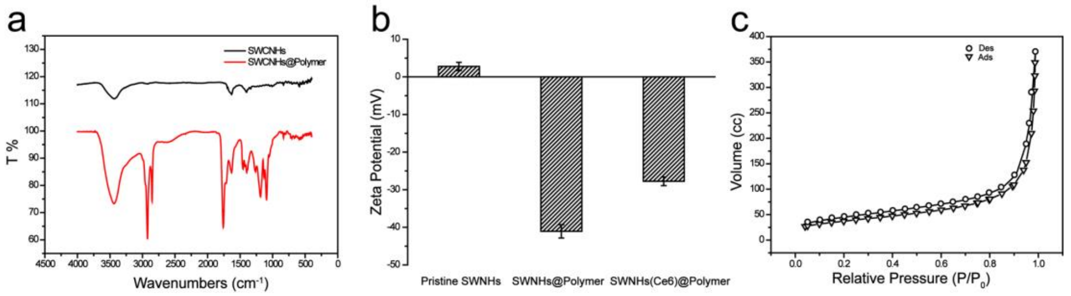


**Figure S1**. Characterization of Gd-Ce6@SWNHs. a) FTIR spectra of pristine SWNHs and Gd-Ce6@SWNHs indicating the polymers were successfully coated onto SWNHs. b) The changes of zeta potential obtained at different synthesis steps of Gd-Ce6@SWNHs. c) BET isotherm measurements of nitrogen adsorption-desorption of Gd-Ce6@SWNHs.

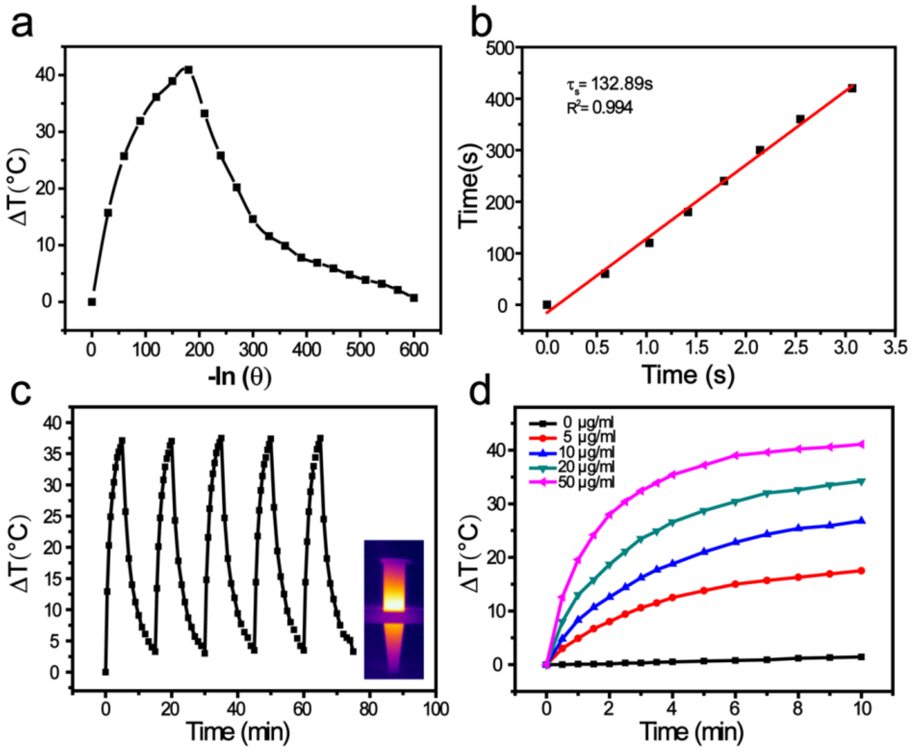


**Figure S2**. Characterization of the photothermal performance of Gd-Ce6@SWNHs. a) Temperature change curve of Gd-Ce6@SWNHs suspensions (400 μL, 50 μg $mL^{-1}$) irradiated by an 808 nm laser with a density of 1 $Wcm^{-2}$ for 180 s. b) Time constant of heat transfer ($\tau_s$) from the system was determined to be 132.89 s by applying the linearized energy balance of temperature *vs* time data from the cooling period of a. c) Photothermal stability of Gd-Ce6@SWNHs evaluated by measuring the real-time temperature of its suspensions (20 μg SWNHs $mL^{-1}$, 400 μL) irradiated by an 808 nm laser (1 W $cm^{-2}$) for five cycles. Each cycle consisted of 3-min laser irradiation followed by a cooling phase. The inset represents infrared thermal photographs of Gd-Ce6@SWNHs suspensions. d) The change of temperature for various concentrations of Gd-Ce6@SWNHs suspensions (0-50 μg $mL^{-1}$) irradiated by an 808 nm laser at 1 W $cm^{-2}$ for 10 min.

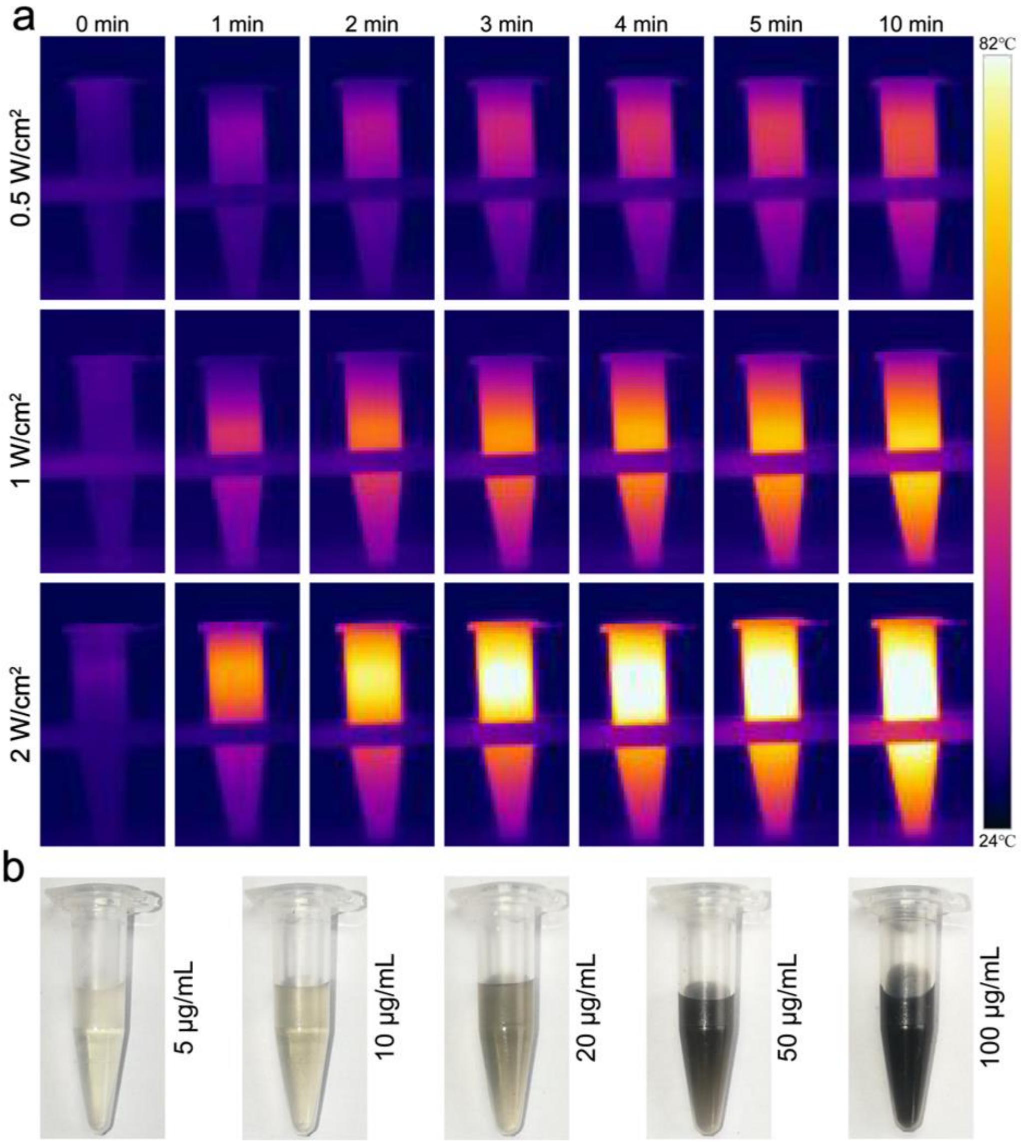


**Figure S3**. The photothermal stability of Gd-Ce6@SWNHs. a) Infrared thermal images of Gd-Ce6@SWNHs suspension (50 μg $mL^{-1}$) exposed to 808 nm laser with the appointed densities of 0.5, 1, and 2 $Wcm^{-2}$ for 10 min. b) Digital photographs of Gd-Ce6@SWNHs at various concentrations (5-100 μg $mL^{-1}$) after the irradiations.

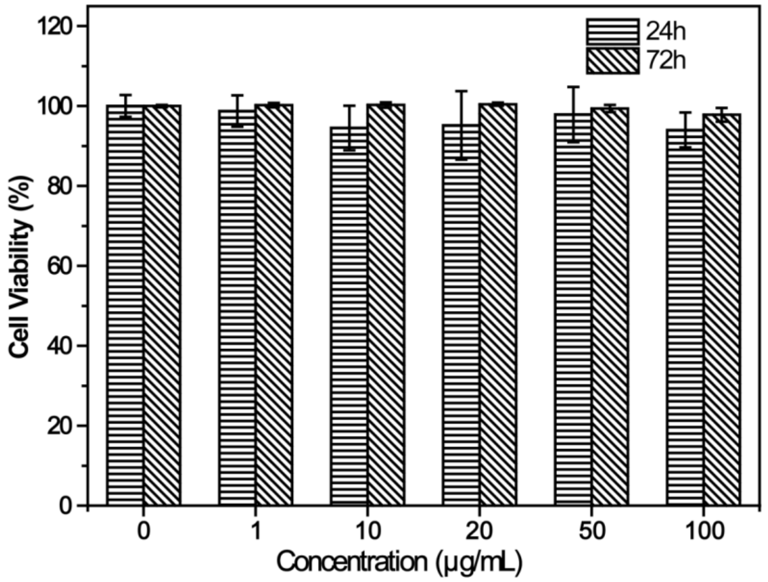


**Figure S4**. Relative cell viabilities of 4T1 cells measured by CCK-8 assay after incubation with Gd-Ce6@SWNHs at various concentrations (0, 1, 10, 20, 50 and 100 μg $mL^{-1}$) for 24 or 72 h. Data were represented as means ± s.d. n = 3.

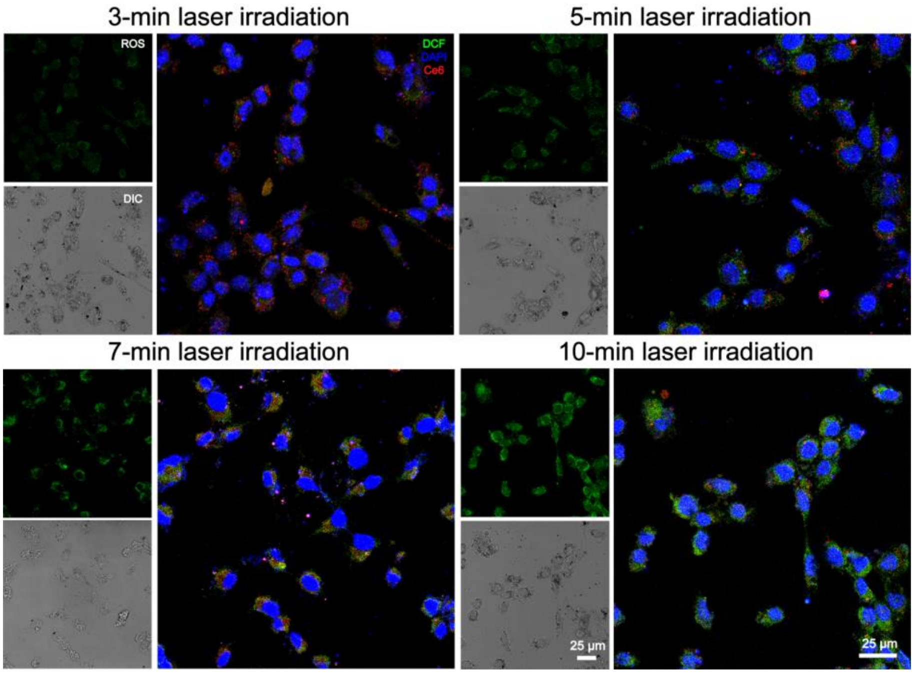


**Figure S5**. Detection of singlet oxygen by the DCFA-HA test after 4T1 cells were co-incubated with Gd-Ce6@SWNHs (10 μg $mL^{-1}$) for 12 h and subsequently exposed to 650 nm light (40 mW $cm^{-2}$) for different periods of time.

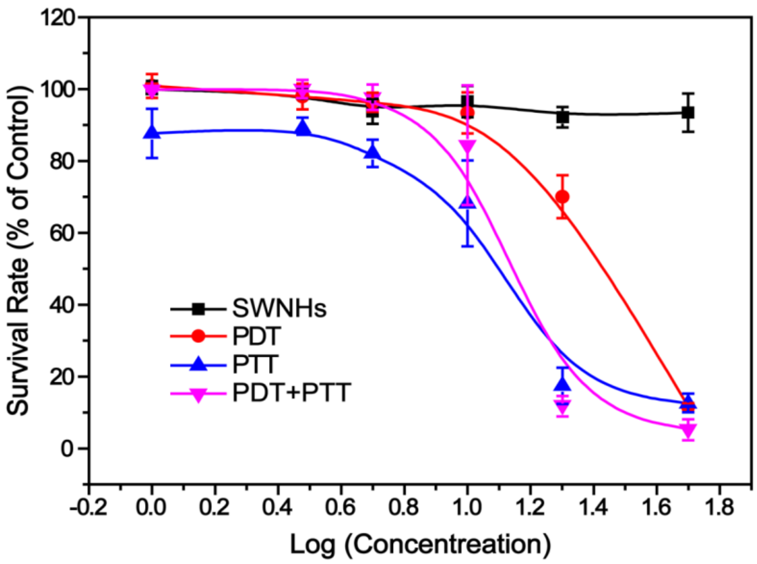


**Figure S6**. The survival rate of 4T1 cells as a function of the logarithm of SWNHs concentration (μg $mL^{-1}$) to calculate the value of $IC_{50}$ according to the fitting curve equations after cells were subjected to various NIR phototherapies.

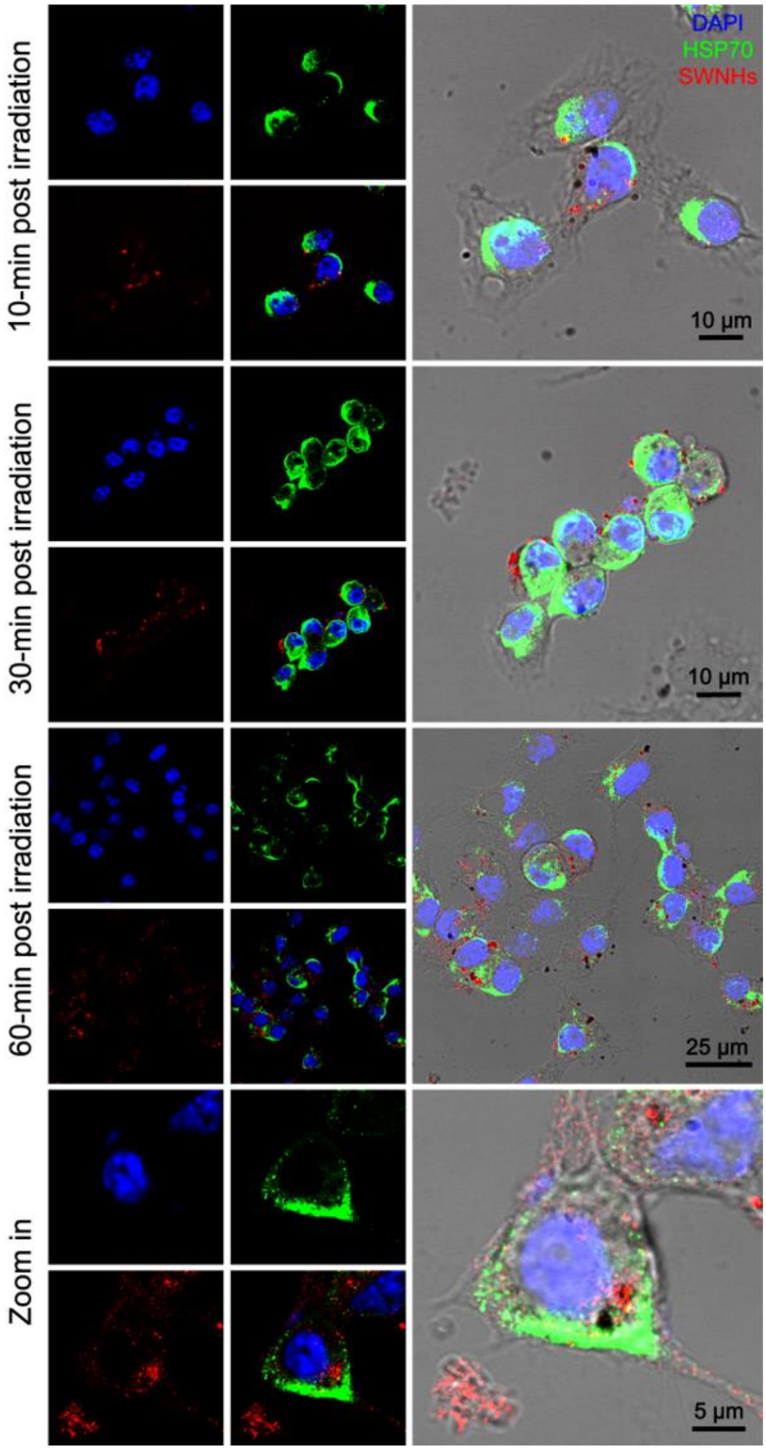


**Figure S7**. The synthesis and migration of heat shock protein (HSP) 70 from nuclei to the cell membrane after the 4T1 cells were cocultured with SWNHs (10 μg $mL^{-1}$) for 12 h and then exposed to 808 nm laser (1.5 W $cm^{-2}$) for 5 min. Tumor cells become bulged and blistering when subjected to heat stress.

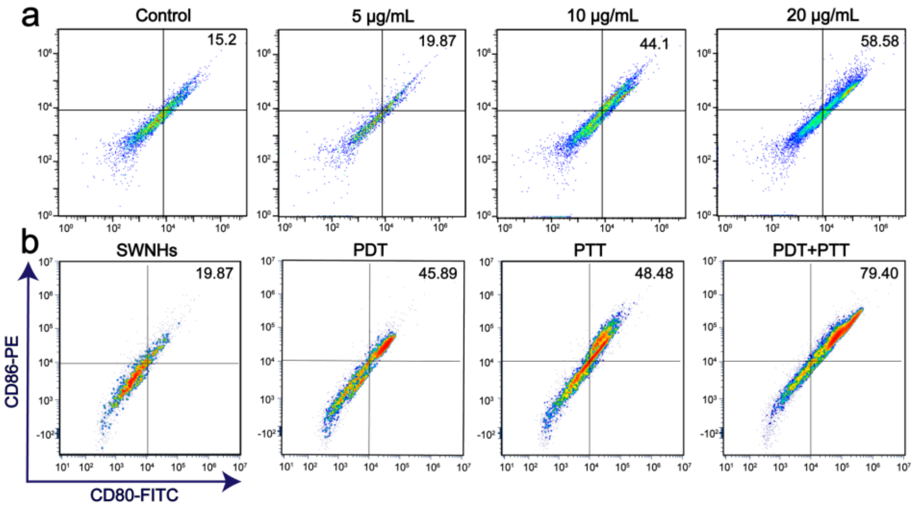


**Figure S8**. Maturation of immature bone marrow-derived DCs co-cultured with various concentrations of Gd-Ce6@SWNHs (0-20 μg $mL^{-1}$) a) or treated with different therapeutics b) using the transwell system.

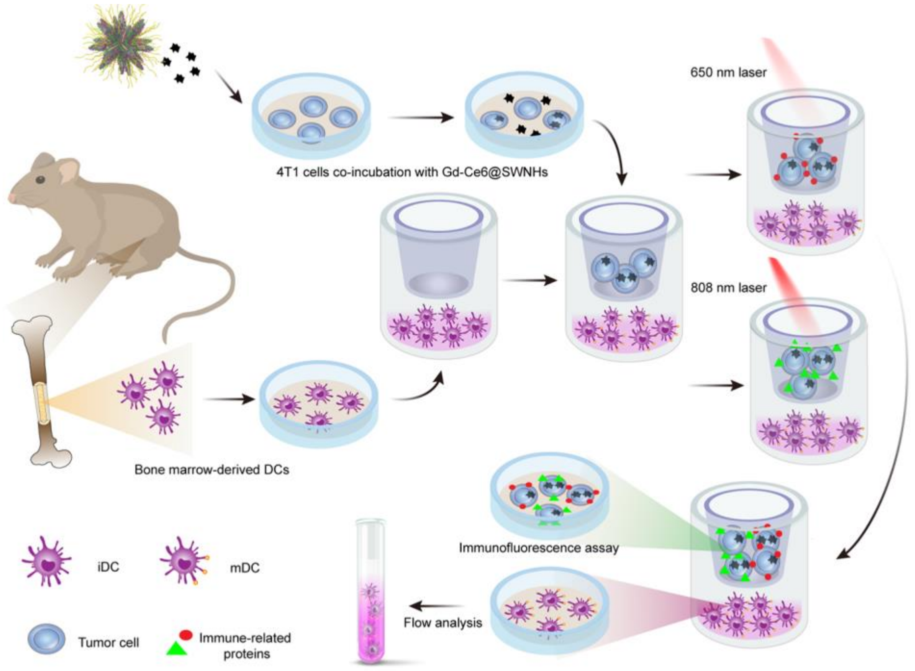


**Figure S9**. The schematic illustration of the process of immunogenicity triggered by NIR phototherapy *in vitro*.

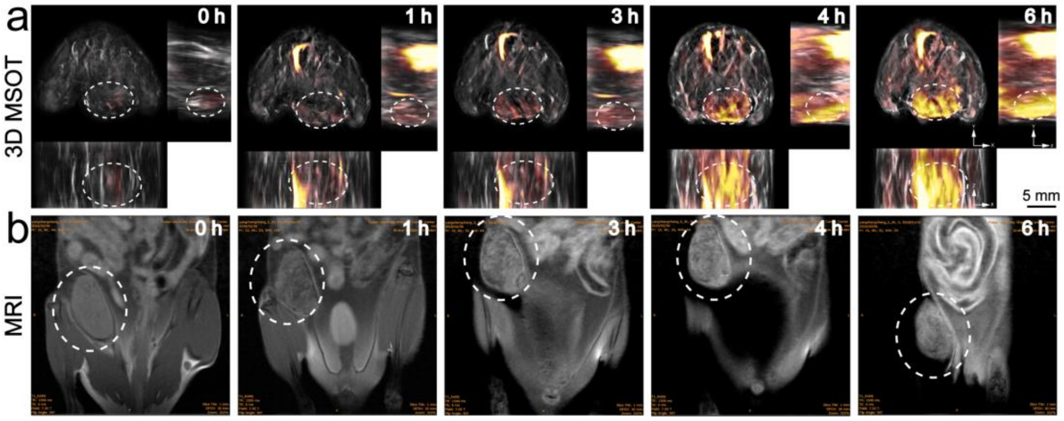


**Figure S10**. a) *In vivo* real-time visualization of Gd-Ce6@SWNHs accumulation in the tumors by 3D MSOT visual modeling after the mice were intravenously injected with Gd-Ce6@SWNHs at a dosage of 10 mg $kg^{-1}$. b) T1-weighted MR imaging of tumors. The circles represented the location of the tumor.

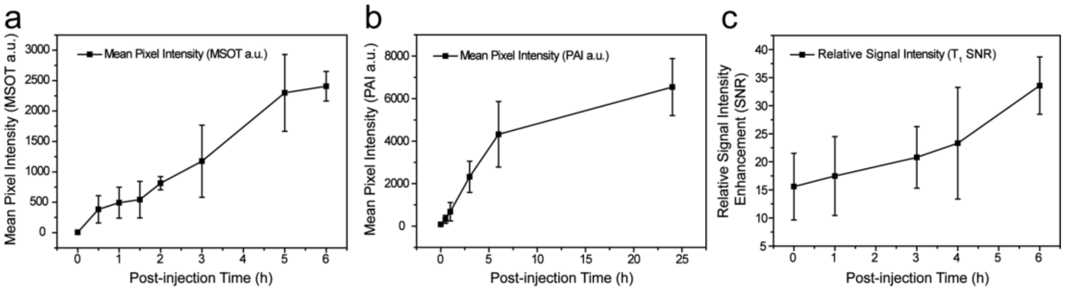


**Figure S11**. Semi-quantitative analysis of Gd-Ce6@SWNHs accumulation in the tumors by a) MSOT, b) PA and c) MRI after the mice were intravenously injected with the Gd-Ce6@SWNHs at a dosage of 10 mg $kg^{-1}$ (in SWNHs) within 24 h.

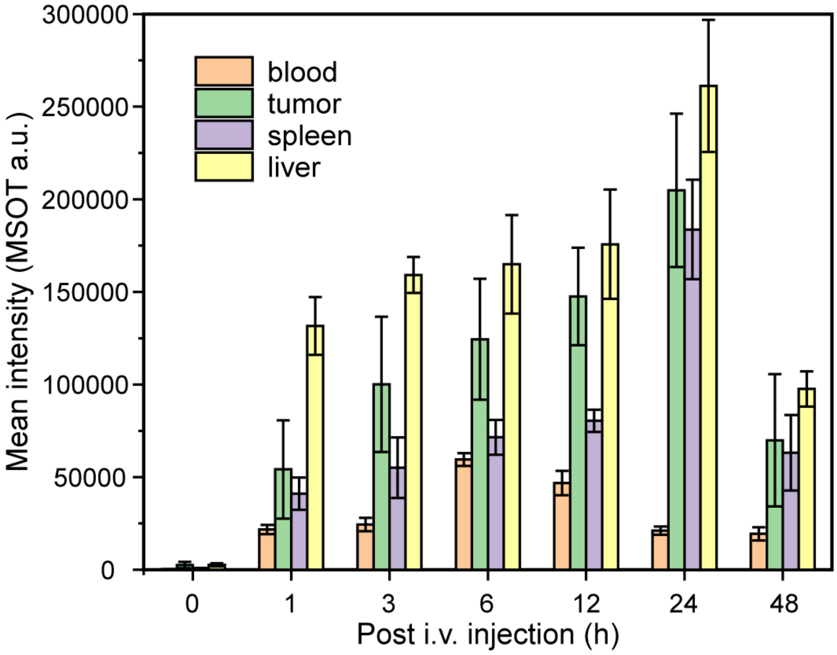


**Figure S12**. Semi-quantitative analysis of the spatial distribution Gd-Ce6@SWNHs in tumor and major organs according to 3D MSOT imaging post 48 h intravenous injection.

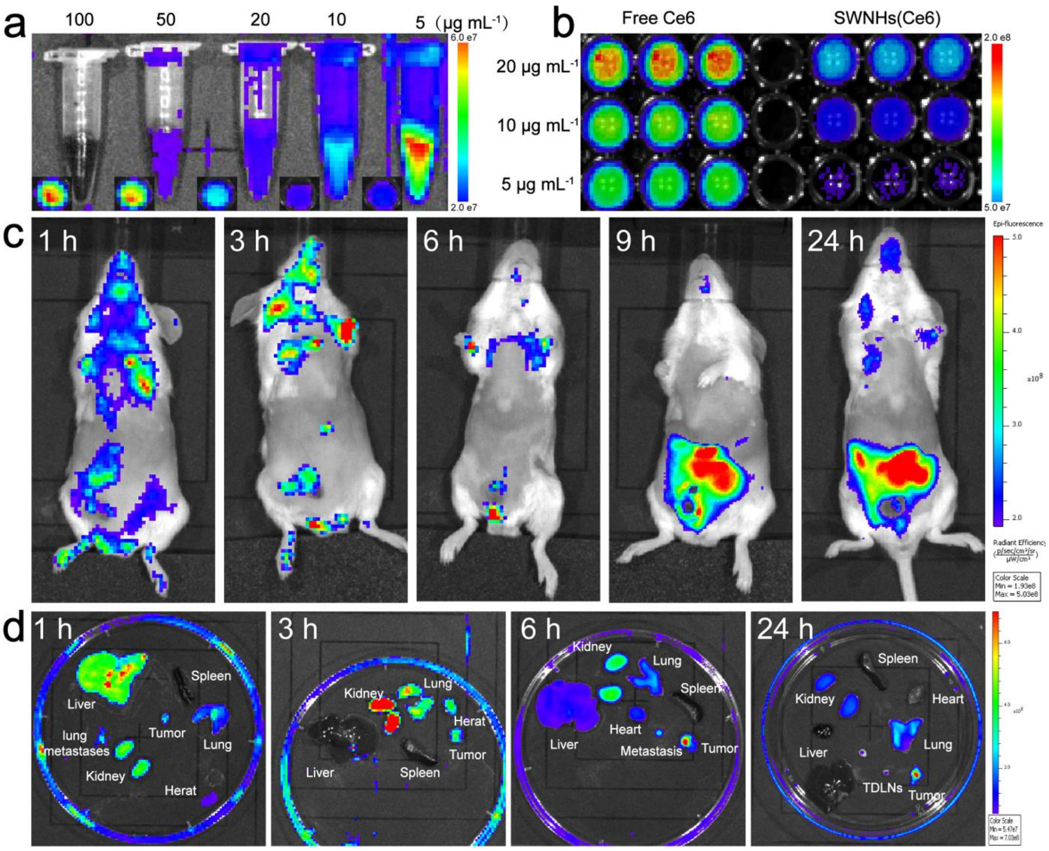


**Figure S13**. a) Detection of the fluorescent signal of Gd-Ce6@SWNHs at different concentrations. The inset showed the corresponding fluorescent signal of free Ce6 that released from Gd-Ce6@SWNHs. b) The fluorescent signal of 4T1 cells after incubation with free Ce6 or Gd-Ce6@SWNHs (at an equivalent concentration of Ce6 from 5-20 μg $mL^{-1}$) for 12 h. c,d), Biodistribution of Gd-Ce6@SWNHs after 1, 3, 6 and 24 h post-injection evaluated by fluorescence imaging c) *in vivo* and d) *ex vivo*.

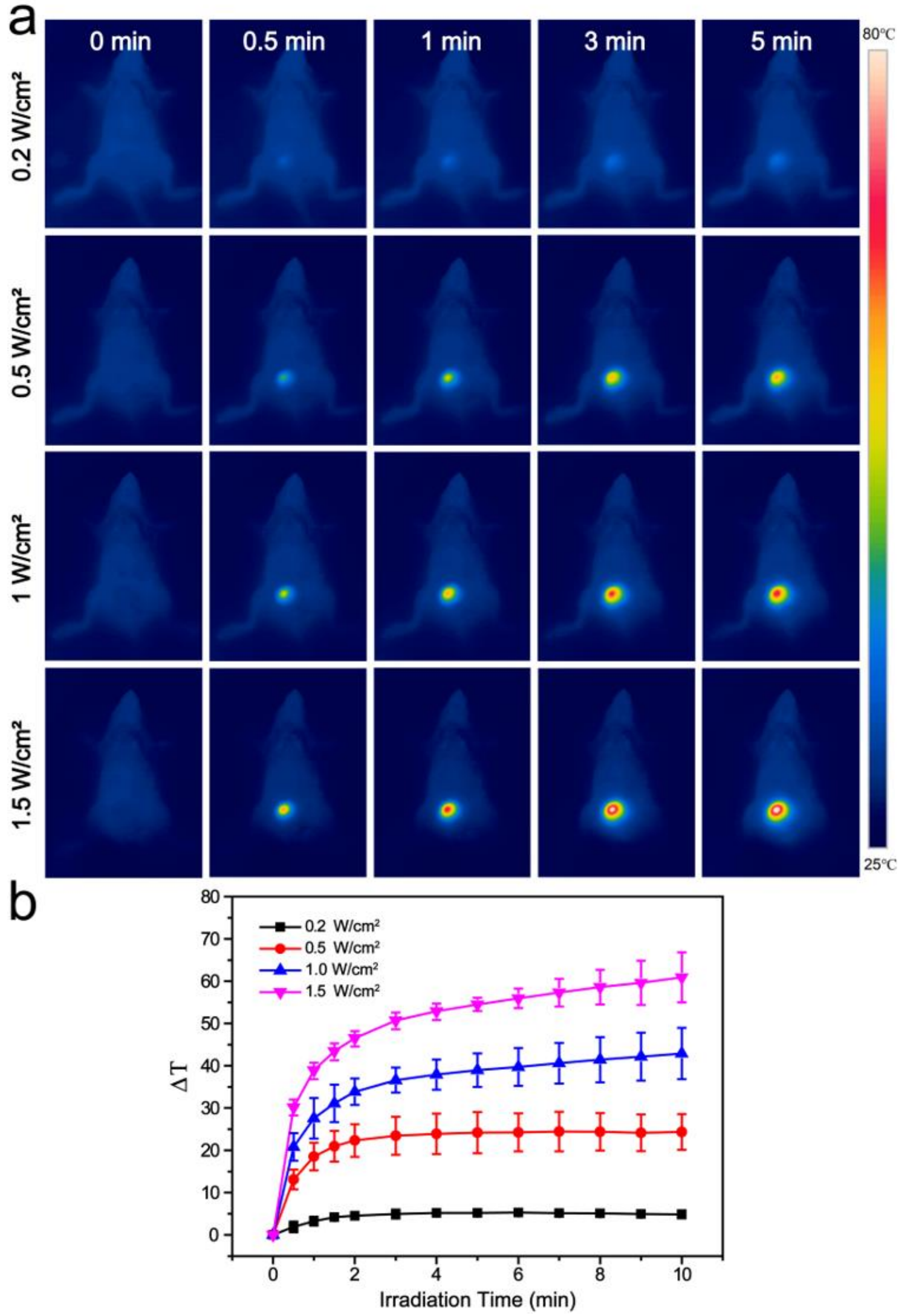


**Figure S14**. Optimization of the laser power intensity and irradiation time for tumor PTT.

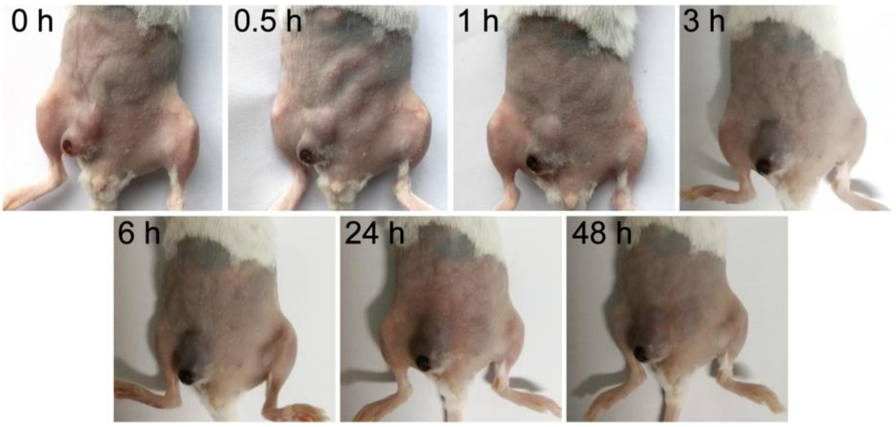


**Figure S15**. Visual inspection of Gd-Ce6@SWNHs accumulation in the primary tumor during 48 h after tumor-bearing mice were intravenously injected with Gd-Ce6@SWNHs at the dosage of 10 mg $kg^{-1}$ b. w.

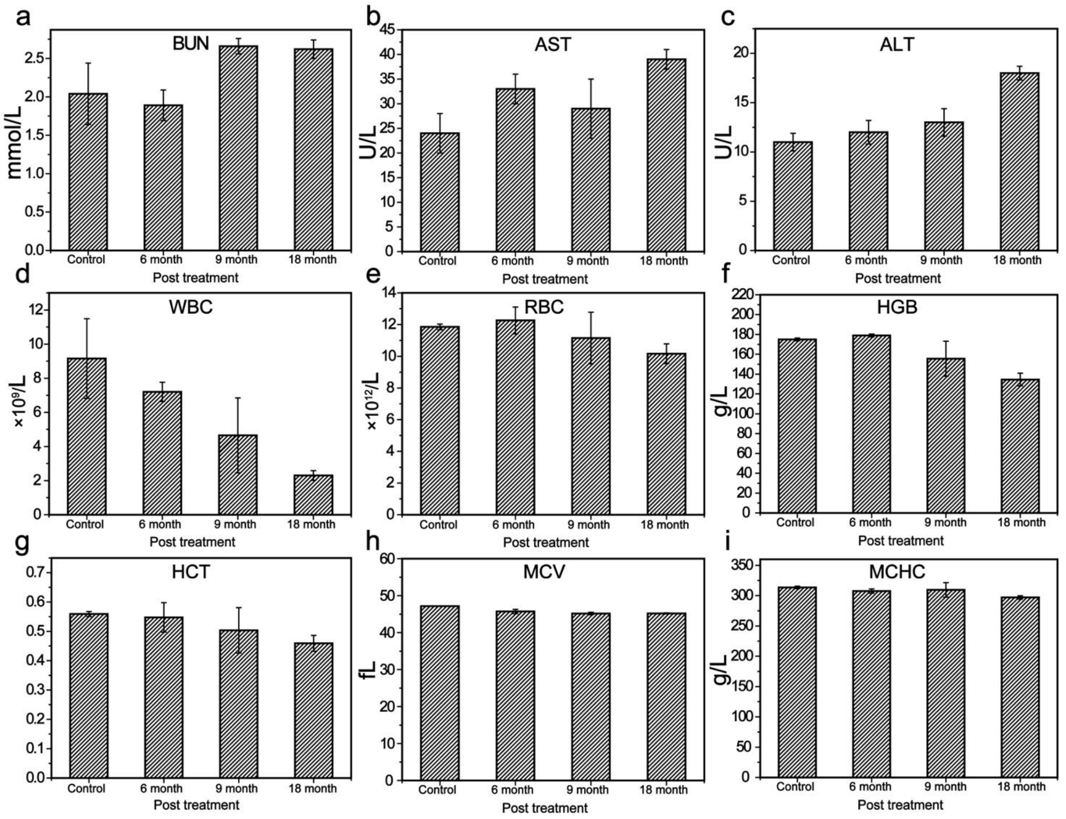


**Figure S16**. Assessment of the long-term toxicity of Gd-Ce6@SWNHs *in vivo*. a-i), Hematological and biochemical parameters of the cured mice sampled from peripheral blood in 6-, 9- and 18-month post-treatment. Six-old normal female mice without any treatment were used as a

negative control. Take samples three times at each determined point for testing. The abbreviations showed in figure a-i) were as follows: blood urea nitrogen (BUN), aspartate aminotransferase (AST), alanine aminotransferase (ALT), white blood cells (WBC), red blood cells(RBC), hemoglobin (HGB), hematocrit (HCT), mean corpuscular volume (MCV), mean corpuscular hemoglobin concentration (MCHC). Data were expressed as means ± s.d.

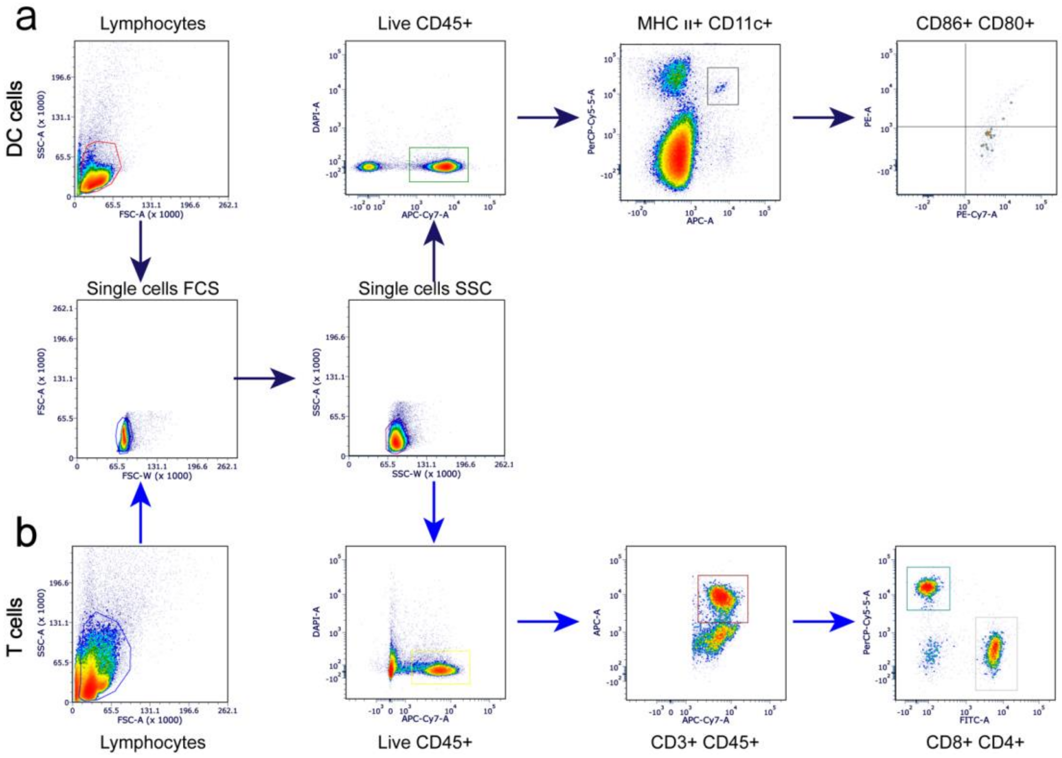


**Figure S17**. Staining gating strategy of DC cells a) and T cells b) applied in flow cytometry to analyze the recruitment of the immune cells into metastatic tumors and tumor-draining lymph nodes.

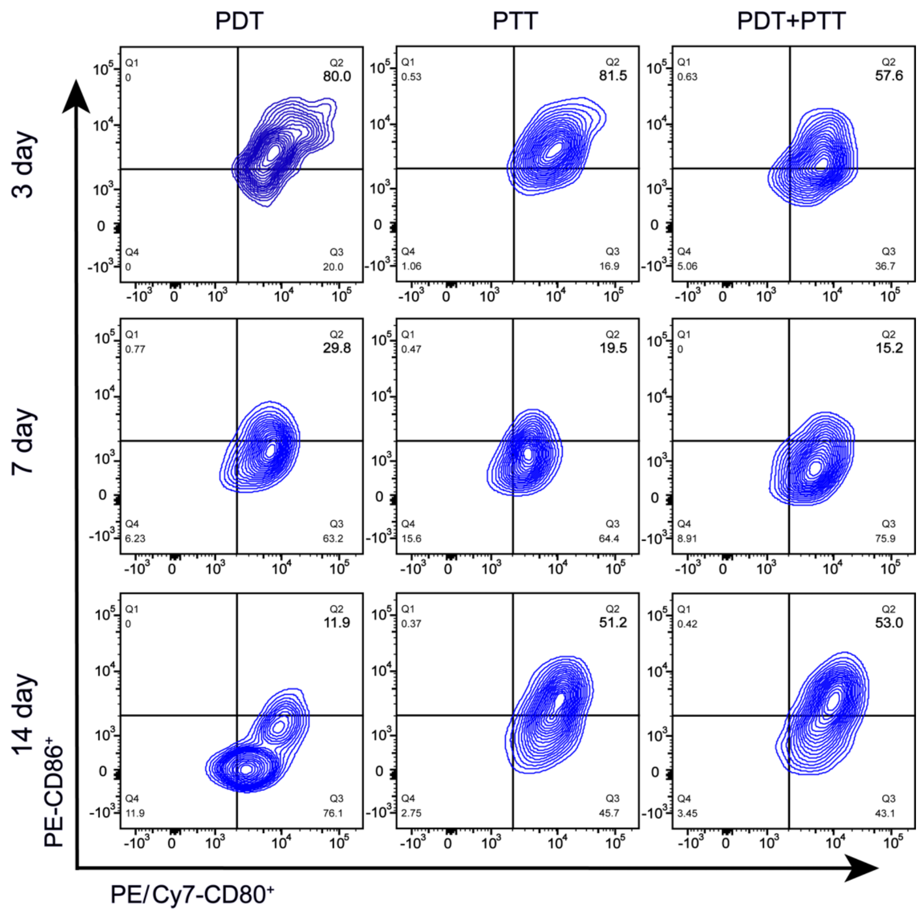


**Figure S18**. Proportions of mature DCs in the distant tumors at day 3,7 and 14 after different phototherapy.

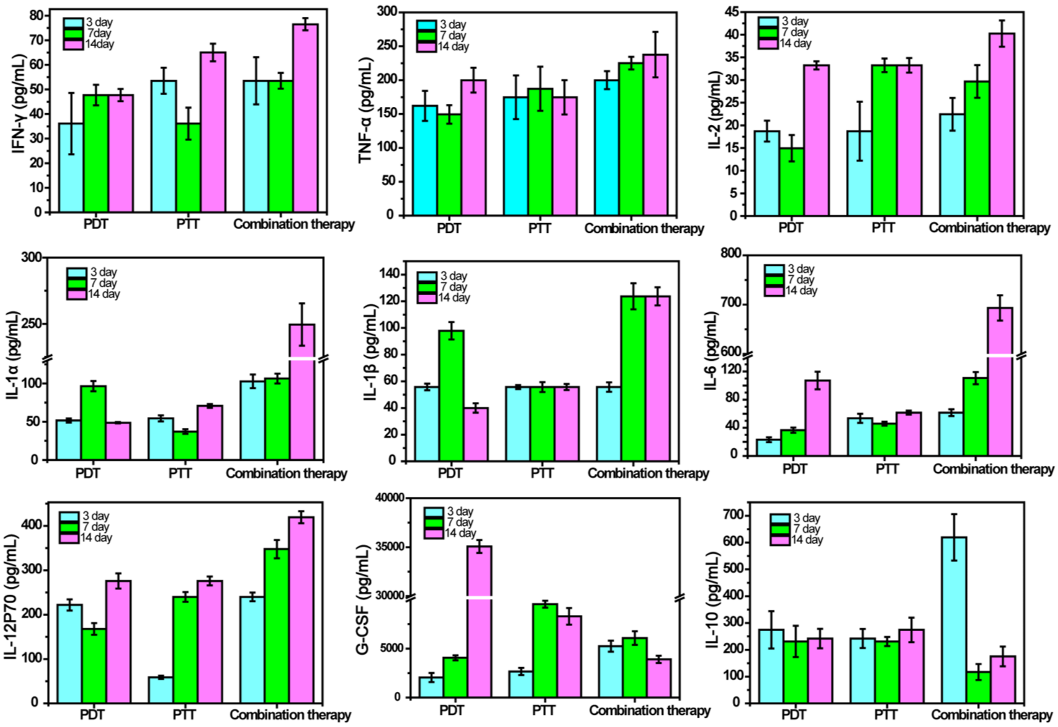


**Figure S19**. Detection of immune-related factors in peripheral serum 3, 7 and 14 days after various treatments. *, $p < 0.05$; **, $p < 0.01$.

**Table 1**. Fold change of gene expression of DCs relative to controls measured by quantitative RT-PCR after various phototherapies*.

| | **SWNHs (10 μg/mL)** | **PTT** | **PDT** | **PTT+PDT** |
|---|---|---|---|---|
| **IFN-γ** | 2.47 | 1.66 | 4.01 | 5.18 |
| **IL-1β** | 43.72 | 0.17 | 1.24 | 2.39 |
| **IL-6** | 85.90 | 15.32 | 3.41 | 232.82 |
| **IL-12** | 2.86 | 0.09 | 0.00 | 0.00 |
| **TNF-α** | 146.68 | 5.56 | 3.55 | 2.75 |
| **CD80** | 54.10 | 0.93 | 5.63 | 8.80 |
| **CD86** | 1.64 | 0.24 | 1.93 | 0.00 |

*: For SWNHs group, DCs were treated with SWNHs; for phototherapy groups, 4T1 cells were treated with SWNHs, and then cocultured with DCs.

**Supporting Information Video 1**. PAI of reconstructed 3D tumor and adjacent tissues before Gd-Ce6@SWNHs injection.

**Supporting Information Video 2**. PAI of reconstructed 3D tumor and adjacent tissues after Gd-Ce6@SWNHs injection for 1 hr.

**Supporting Information Video 3**. PAI of reconstructed 3D tumor and adjacent tissues after Gd-Ce6@SWNHs injection for 3 hrs.

**Supporting Information Video 4**. PAI of reconstructed 3D tumor and adjacent tissues after Gd-Ce6@SWNHs injection for 6 hrs.

**Supporting Information Video 5**. 3D MSOT imaging of whole body after Gd-Ce6@SWNHs injection for 1 hr.

**Supporting Information Video 6**. 3D MSOT imaging of whole body after Gd-Ce6@SWNHs injection for 3 hrs.

**Supporting Information Video 7**. 3D MSOT imaging of whole body after Gd-Ce6@SWNHs injection for 6 hrs.

**Supporting Information Video 8**. 3D MSOT imaging of whole body after Gd-Ce6@SWNHs injection for 24 hrs.
